# Socio-environmental assessment of *Aedes aegypti* distribution and risk of chikungunya in vulnerable community

**DOI:** 10.1371/journal.pntd.0014776

**Published:** 2026-10-12

**Authors:** Jaqueline S. Cruz, Hernan D. Argibay, Nivison Nery, Hammed Mogaji, Thiago da M. Barreto, Lorena Ellen S. Batista, Lara R. S. Silva, Victor Kaua Nery, Victoria C. Dedavid Ferreira, Ianei de O. Carneiro, Yeimi A. Alzate López, Mitermayer G. Reis, Albert I. Ko, Petter Diggle, Michael Begon, Federico Costa, Hussein Khalil

**Affiliations:** 1 Instituto Gonçalo Moniz, Fundação Oswaldo Cruz, Ministério da Saúde, Salvador, Bahia, Brazil; 2 Instituto de Saúde Coletiva, Universidade Federal da Bahia, Salvador, Bahia, Brazil; 3 Department of Epidemiology of Microbial Diseases, Yale School of Public Health, New Haven, Connecticut, United States of America; 4 Escola de Medicina Veterinária e Zootecnia, Universidade Federal da Bahia, Salvador, Bahia, Brazil; 5 Faculdade de Medicina, Universidade Federal da Bahia, Salvador, Bahia, Brazil; 6 Centre for Health Informatics, Computing, and Statistics, Lancaster University, Lancaster, United Kingdom; 7 Department of Evolution, Ecology and Behaviour, The University of Liverpool, Liverpool, United Kingdom; 8 Department of Wildlife, Fish, and Environmental Studies, Swedish University of Agricultural Sciences, Umeå, Sweden; Egerton University, KENYA

## Abstract

Chikungunya virus (CHIKV), transmitted by *Aedes* mosquitoes, poses a major public health challenge in tropical regions. In Brazil, its incidence has increased, especially in socioeconomically vulnerable areas, where environmental conditions favor the proliferation of vectors. Our objective was to assess the socioeconomic and environmental vulnerabilities associated with the spatial distribution of a) *Ae. aegypti* and b) resident exposure to CHIKV in a low-income community. Identifying key exposure hotspots, our work improves our understanding of CHIKV transmission in informal settlements and facilitates community-based interventions to mitigate CHIKV risk. We monitored *Ae. aegypti* using ovitraps and conducted a serological survey from October 2021 to June 2022 in a vulnerable informal community in Salvador, Brazil. Blood samples and sociodemographic, household, and environmental data were collected from residents aged ≥5 years. CHIKV serology was performed, and spatial models using Integrated Nested Laplace Approximation assessed vector distribution, seropositivity, and associated factors. Among 194 ovitraps, posterior estimates indicated higher egg counts in peridomicile locations (IRR = 1.99; CrI:1.31-3.06); households experiencing food insecurity (IRR = 1.77; CrI:1.05 - 2.99), properties with unpaved yards (IRR = 3.76; CrI:2.31-6.18), and areas with greater garbage density (IRR = 1.25; CrI:1.01 -1.56). Among 888 participants, Bayesian spatial modeling identified factors associated with CHIKV seropositivity: elderly (OR= 2.24; CrI:1.16-4.35), uncovered brick walls (OR=2.29; CrI:1.16-4.55), the presence of yards, either paved (OR=2.06; CrI:1.30-3.00) or unpaved (OR=2.71; CrI:1.73-4.25), and self-reported mosquito sightings (OR=1.94; CrI:1.19-3.18). In addition, remotely sensed Land Surface Temperature (OR=1.33; CrI:0.98-1.83) and the spatial ovitraps egg counts (OR=1.14; CrI:0.87-1.52) were relevant variables. Our high-resolution study identified fine-scale vulnerabilities linked to both *Ae. aegypti* abundance and heightened risk of CHIKV transmission. Based on our findings, effective control strategies should not only focus on traditional methods such as enhancing basic urban infrastructure and services and promoting community awareness, but also target specific breeding sites around homes, address garbage accumulation, and consider the influence of micro-scale temperature variations. These results emphasize the importance of tailored, neighborhood-specific interventions to effectively reduce chikungunya transmission in high-risk communities.

## Author summary

Chikungunya virus (CHIKV) infection is an increasing public health concern in Brazil, particularly in socioeconomically vulnerable urban communities where environmental conditions favor the proliferation of *Aedes* mosquitoes. Despite this, much of the existing evidence on CHIKV transmission relies on aggregated data at neighborhood or city scales, which may overlook important heterogeneity in exposure risk within informal settlements. Given the strong influence of household conditions and local environments on mosquito breeding and virus transmission, fine-scale investigations are essential to better understand CHIKV dynamics in these settings. In this study, we examined the spatial distribution of *Aedes aegypti* and human exposure to CHIKV in a low-income community in Salvador, Brazil. We conducted entomological surveillance using ovitraps and a serological survey among community residents, alongside the collection of sociodemographic, household, and environmental information. By integrating high-resolution field data with spatial modeling approaches, this study aimed to characterize how social and environmental vulnerabilities shape mosquito distribution and CHIKV exposure at small spatial scales. This work seeks to inform more precise, locally tailored strategies for chikungunya prevention and control in vulnerable urban communities.

## Introduction

Chikungunya (CHIKV), an arboviral disease, has emerged as a significant public health concern in tropical and subtropical regions in recent decades [1]. Since its identification in Tanzania in 1952, the virus has spread to large regions of Africa, Asia, Oceania, and the Americas, with sporadic outbreaks reported in these areas over the years [1–3]. Cumulative global cases of CHIKV infection are estimated to have reached 10 million [4], with approximately 1.3 billion people living in regions at high risk for transmission [5]. In Brazil, since its first detection in 2014, chikungunya has spread rapidly, causing sporadic outbreaks across the country, affecting thousands of people, and posing significant challenges to healthcare systems and vector control efforts [6,7]. CHIKV typically causes acute fever and severe joint pain, with possible chronic arthritis and rare complications like neurological or cardiac issues, primarily affecting older adults and vulnerable populations [1,8]. Transmission of CHIKV occurs primarily through the bite of female mosquitoes of the *Aedes* genus, particularly *Ae. aegypti* and *Ae. albopictus* [8], which are widely distributed in urban environments. The proliferation of these mosquitoes is driven by a combination of environmental and socioeconomic factors commonly observed in low-income and informal communities [9]. Across diverse settings, prior syntheses of the evidence indicate that structural deprivation and features of the urban environment consistently shape *Aedes* mosquito distribution and arboviral disease risk [10]. In northeastern Brazil, where the annual mean temperature is approximately 27°C (mean minimum 25°C and mean maximum 28°C), the increase in both natural and anthropogenic breeding sites is associated with water accumulation and improper water storage practices, together with an increased rainfall during the first half of the year [11]. Seasonal peaks in vector abundance are epidemiologically significant as they tend to precede or coincide with increased transmission of arboviruses such as dengue, chikungunya, and Zika [12], highlighting the importance of integrating climatic drivers into surveillance and control strategies in endemic urban settings. Additionally, climate conditions, high vector density, extensive movement of individuals, and the presence of people susceptible to infection are relevant factors contributing to the high number of cases [13,14].

While previous studies have explored the relationship between environmental factors and the distribution of diseases like chikungunya [5,15–17], there remains a lack of comprehensive, community-level assessments integrating both socio-economic and environmental variables influencing both vector proliferation and human exposure. Understanding the interaction between environmental factors and disease distribution is essential for public health management, particularly in vulnerable communities. Integrated policies are needed to promote social mobilization, health and environmental education, access to adequate housing and sanitation, and prevent deforestation [18,19].

Here, we combine environmental, demographic, socioeconomic, and entomological data at a fine scale to identify risk factors and hotspots for *Ae. aegypti* breeding and CHIKV exposure. Specifically, the aim of the present study was to evaluate the factors associated with the spatial distribution of *Ae. aegypti* and the exposure of residents to chikungunya in a low-income community in the city of Salvador, Brazil.

## Results

The demographic and exposure information of all the participants included in the study is summarized in Table 1 together with the bivariate GLMM result. Among the participants, there was no significant difference in exposure in relation to sex, race, presence of running water in the home, water storage in the home, paved home entry, or observed mosquitoes in the home. However, certain factors showed a significant association with exposure, such as advanced age (≥65 years) (p = 0.02), stable union or legally married (p = 0.04), education level (p = 0.019), uncovered brick or non-masonry construction (p = 0.02), and lack of a yard (p < 0.001).

**Table 1:** Individual- and household-level characteristics according to chikungunya virus serological status and results from univariable analyses.

Characteristic	Total N = 888	Negatives N = 513 (58%)	Positives N = 375 (42%)	p-value
**Individual characteristic**				
Sex				
Female	540	314 (58)	226 (42)	
Male	348	199 (57)	149 (43)	0.9
Age (years)				
5–17	183	113 (62)	70 (38)	
18–44	397	242 (61)	155 (39)	0.93
45–64	213	113 (53)	100 (47)	0.069
Above 65	84	40 (48)	44 (52)	**0.022**
Self-reported skin color				
Black or mixed-race	456	456 (57)	339 (43)	
Others	19	11 (58)	8 (42)	0.83
White	70	43 (61)	27 (39)	0.52
Marital Status				
Single	571	344 (60)	227 (40)	
Stable union or legally married	251	132 (53)	119 (47)	**0.04**
Separated, divorced or widowed	63	35 (56)	28 (44)	0.42
Education (years)				
0–9	488	265 (54)	223 (46)	
> 9	397	246 (62)	151 (38)	**0.019**
**Household characteristic**				
Running water in the home				
No	39	21 (54)	18 (46)	
Yes	831	480 (58)	351 (42)	0.93
Water storage in the home				
No	185	112 (61)	73 (39)	
Yes	682	386 (57)	296 (43)	0.3
Not sure	3	3 (100)	0 (0)	>0.99
Paved home entry				
Yes	835	483 (58)	352 (42)	
No	35	18 (51)	17 (49)	0.45
Wall material				
Plastered wall	810	476 (59)	334 (41)	
Uncovered brick or other than masonry	60	25 (42)	35 (58)	**0.02**
Yard type				
Unpaved yard	211	99 (47)	112 (53)	
Paved yard	218	115(53)	103 (47)	0.4
No yard	441	287 (65)	154 (35)	**<0.001**
Observed mosquito in your home (<7 days)				
Yes	714	400 (56)	314 (44)	
No	156	101 (65)	55 (35)	0.06

Bold values indicate statistically significant results (p < 0.05).

Regarding the ovitraps results, a total of 194 ovitraps were successfully collected after 5–7 days, of which 134 were positive (69.1%), with 56/99 (56.6%) positives within the intradomicile and 78 /95 (82.1%) in the peridomicile. The overall mean number of eggs collected per ovitrap was 49.1, with a median of 13.5 (IQR: 0 – 60). The final INLA-SPDE model retained ovitrap loca tion, type of yard, food insecurity and garbage accumulation points density (S1 Table). The data from the final model on egg counts in the ovitraps are shown in Table 2. The model indicated higher egg counts in ovitraps placed in the peridomicile (area around homes) compared to those placed in the intradomicile (IRR = 1.99; credible interval (CrI): 1.25 - 3.06). Similarly, the lack of pavement in the yard (unpaved yard) was associated with substantially higher egg counts (IRR = 3.76; CrI: 2.31 - 6.18). Egg counts also increased with the density of garbage accumulation points within the community (IRR = 1.25; CrI: 1.01 - 1.56), suggesting localized environmental influences on mosquito oviposition. In addition, food insecurity was positively associated with the egg count (IRR = 1.77; CrI: 1.05 -2.99).

**Table 2:** Results from the INLA-SPDE model for *Ae. aegypti* egg count in ovitrapsovitraps.

Predictive variable	IRR
Ovitrap loca tion	
Intradomicile	ref
Peridomicile	**1.99 (1.31 -** **3.06)**
Food insecurity	
No	ref
Yes	**1.77 (1.05 - 2.99)**
Premise type of yard	
No yard	ref
Paved yard	1.36 (0.81 - 2.29)
Unpaved yard	**3.76 (2.31 - 6.18)**
Garbage points density	**1.25 (1.01 -** **1.56**)

Bold values indicate incidence rate ratios with 95% credible intervals excluding 1.

The final INLA-SPDE model for chikungunya exposure outcome retained age, wall material, type of yard, self-reported mosquito sightings, water storage practices, land surface temperature and the spatially predicted ovitrap egg counts as explanatory variables (S2 Table). Posterior estimates indicated higher odds of CHIKV seropositivity among individuals aged ≥65 years (OR = 2.24; CrI: 1.16 – 4.35) and residents of households with uncovered brick walls (OR = 2.29; CrI: 1.16 – 4.55). The presence of a yard was also associated with increased odds of seropositivity, whether the yard was paved (OR = 2.06; CrI: 1.31 – 3.30) or unpaved (OR = 2.71; CrI: 1.74 – 4.25), compared with absence of yards. In addition, self-reported mosquito sightings (OR = 1.94; CrI: 1.19 – 3.17) and water storage practices (OR: 1.59; CrI: 1.00 - 2.56) were positively associated with CHIKV seropositivity. Land surface temperature and spatial egg counts were included in the final model because they marginally improved model fit and enhanced biological interpretability. Although their 95% credible intervals included the null value, differences in WAIC among competing models were negligible (<2), indicating comparable predictive performance. These variables were therefore retained to capture ecologically relevant processes potentially linking environmental conditions, vector abundance, and CHIKV transmission (Table 3).

**Table 3:** Final multivariate model of chikungunya prevalence in the human population assessing spatial dependency.

	OR (95% CI)
Age	
5 - 20	ref
21 - 44	1.06 (0.71 - 1.58)
45 - 64	1.36 (0.86 - 2.14)
≥ 65	**2.24 (1.16 - 4.35)**
Wall material	
Plastered wall	ref
Uncovered brick or other than masonry	**2.29** **(1.16 - 4.55)**
Yard type	
Not yard	ref
Paved yard	**2.06** **(1.30 - 3.00**)
Unpaved yard	**2.71 (1.73 - 4.25**)
Self-reported mosquito sightings	
No	ref
Yes	**1.94 (1.19 - 3.17)**
Water storage practice	
No	ref
Yes	1.94 (1.00 - 2.56)
Land Surface Temperature	1.33 (0.98 - 1.83)
Egg count spatial prediction	1.14 (0.87 - 1.52)

Bold values indicate odds ratios with 95% credible intervals excluding 1.

The environmental assessment results indicated variations in waste concentrations in the study area. The density of litter points is the highest (0 - 4.49), followed by the density of garbage points (0 - 3.23), and the density of building materials points (0 - 2.26) (Fig 1f, 1g and 1h).

**Fig 1:**
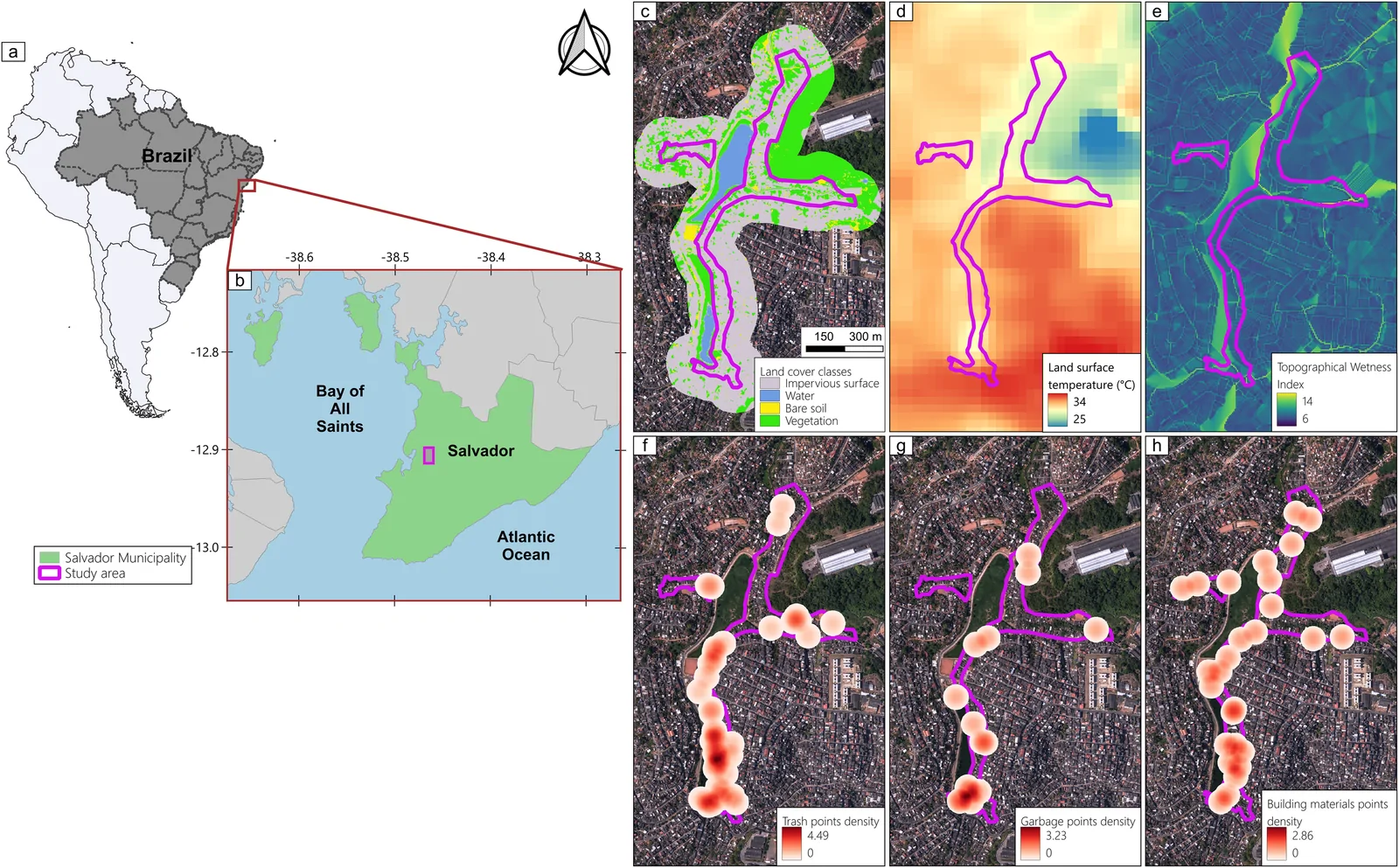
Study area descriptive maps. a) Localization of Salvador city; b) Salvador municipality and location of study area; c) Land cover map of study area. d) Land surface temperature within study area and surroundings; e) Topographical Index Map within study area and surroundings; f, g and h) Kernell density map of trash, garbage and building materials accumulation points, respectively. Spatial vector data of a and b figures were obtained from the Brazilian Institute of Geography and Statistics [20] (IBGE, 2022) based on the Continuous Cartographic Base of Brazil at 1:250,000 scale (BC250, version 2025), available at: https://www.ibge.gov.br/geociencias/downloads-geociencias.html?caminho=cartas_e_mapas/bases_cartograficas_continuas/bc250/versao2025/ (accessed July 2025). Terms of use and access conditions for IBGE geospatial data are available at: https://www.ibge.gov.br/geociencias/downloads-geociencias.html and https://www.ibge.gov.br/acesso-informacao/uso-de-dados.html. IBGE cartographic data are publicly available federal datasets and may be used and reproduced with proper source attribution, in accordance with Brazilian open data policy. The high-resolution base orthoimage used for map visualization was obtained from the Salvador Municipality cartographic services via its WMS service available at https://www.sefaz.salvador.ba.gov.br/geosalvador/home/item.html?id=139b22a13a654f779bbbbd8cf350347d (accessed February 2026). The WMS is provided as publicly accessible municipal open data for consultation and reuse, subject to source attribution in accordance with the municipality’s open data and public information policies (https://www.sefaz.salvador.ba.gov.br/geosalvador/home/item.html?id=a455c391abc4495b9ce96693c4f288b6).

Regarding the spatial distribution, we observed that predicted egg counts per trap ranged from 35 to 127 eggs, with localized areas of high counts, representing a baseline infestation scenario and potential hotspots. The right panel illustrates seroprevalence values ranging from 0.16 to 0.74, highlighting marked spatial variability in the population's exposure to the virus (Fig 2).

**Fig 2:**
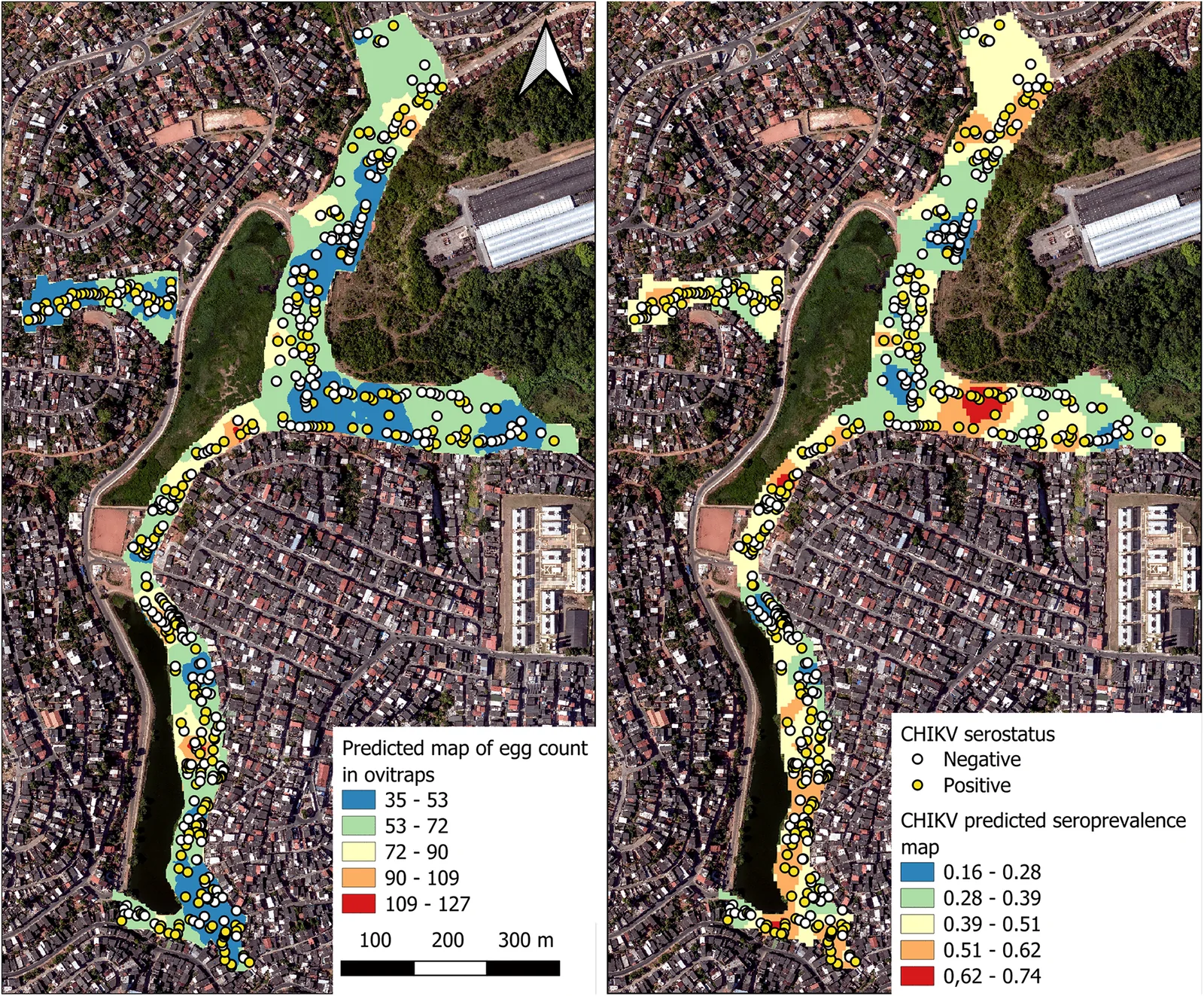
Null smoothed map of *Aedes aegypti* egg counts in ovitraps (left) and seroprevalence map (right). The high-resolution base orthoimage used for map visualization was obtained from the Salvador Municipality cartographic services via its WMS service available at https://www.sefaz.salvador.ba.gov.br/geosalvador/home/item.html?id=139b22a13a654f779bbbbd8cf350347d (accessed February 2026). The WMS is provided as publicly accessible municipal open data for consultation and reuse, subject to source attribution in accordance with the municipality’s open data and public information policies (https://www.sefaz.salvador.ba.gov.br/geosalvador/home/item.html?id=a455c391abc4495b9ce96693c4f288b6).

## Discussion

Our results demonstrate that chikungunya risk in vulnerable communities is structured by the interaction between socio-demographic, environmental, and entomological factors at a micro-spatial scale. By integrating high-resolution entomological, environmental, and serological data within a single informal settlement, we reveal heterogeneity that is obscured in neighborhood- or city-level analyses and provide actionable insights for targeted, community-based interventions.

The seroprevalence of CHIKV observed in our study (42%) is high and consistent with previous reports from Salvador and other urban areas in Brazil following major epidemic waves. Municipality-level analyses in Salvador have documented substantial cumulative exposure with pronounced spatial heterogeneity across neighborhoods [21]. Similarly, seroepidemiologic surveys conducted in other Brazilian regions have reported high levels of prior CHIKV exposure in vulnerable urban settings, often exceeding 30–40% in communities heavily affected by outbreaks [22,23]. These comparisons underscore the substantial and uneven burden of chikungunya in informal settlements.

The analysis revealed a higher egg count of *Ae. aegypti* observed in peridomestic areas, indicat ing that the space surrounding households constitutes a critical interface between environmental vulnerability and vector proliferation. Rather than simply reinforcing the well-established importance of peridomestic environments in vector control, our findings refine this paradigm by demonstrating how fine-scale heterogeneity within a single informal settlement structures entomological risk at distances of only a few tens of meters. In informal urban settings, this area concentrates exposed containers and water accumulation around houses, representing high-risk sites for vector proliferation. This observation was also described by Marina *et al* (2021) in Mexico, and similarly by Sasmita *et al* (2021) who reported that, *Ae. aegypti* was predominantly found in homes and outdoor ovitraps had a higher likelihood of containing mosquito eggs than indoor ones [24,25]. Together, these findings suggest that peridomestic environments may represent a risk for sustaining vector reproduction and could contribute to maintaining transmission even when indoor control measures are implemented. Yard characteristics further modulated vector abundance, with unpaved yards being strongly associated with higher densities of *Ae. aegypti*. The spatial distribution of eggs in urban environments is significantly influenced by microclimatic conditions, with a marked preference for shaded areas and unpaved peridomestic settings [26–28]. These microenvironments act as thermal refuges, preventing water temperature from reaching lethal thresholds for larvae and reducing the evaporation rate within breeding sites [29]. Furthermore, unpaved backyards characterized by natural soil and vegetation contribute to higher relative humidity and the accumulation of organic matter. The decomposition of this organic debris generates volatile chemical cues that are highly attractive to gravid females, signaling a nutrient-rich environment for larval development [30]. As a result, ovitraps placed in these specific niches consistently yield a higher egg density compared to those in exposed or fully paved areas. These findings suggest that targeted control actions focused on the peridomestic environment, such as cleanup campaigns and the elimination of breeding sites, may be effective in reducing vector density in affected areas. However, clean-up campaigns and environmental management interventions did not show the consistent or sustained reductions in *Ae. aegypti* populations that were expected in rigorous trials, underscoring the need for more robust or innovative strategies to achieve meaningful vector suppression and reduced disease transmission [12]. Therefore, rather than reinforcing a purely individual- or household-centered approach, our study supports the need for integrated interventions that combine community engagement with improvements in essential urban services. Strengthening waste management systems and water and sanitation infrastructure is critical for enabling sustainable household-level prevention and tackling the broader environmental and socioeconomic conditions that drive vector abundance.

The association between food insecurity and increased egg counts (IRR = 1.72) underscores the socio-environmental nature of *Ae. aegypti* proliferation. This finding aligns with the well documented theory that vector density is closely tied to ‘structural vulnerability” where households experiencing food instability are likely subject to inadequate sanitation and precarious water storage [9], even within low-income communities. T he stronger association observed for food insecurity over other socioeconomic indicators like income suggests that specific household-level deprivation is a more sensitive predictor of mosquito abundance than absolute income alone. This aligns with the ‘Social Producers of Disease’ framework, which posits that the lack of resources limits a household’s capacity to eliminate cryptic breeding sites, thereby increasing local entomological risk [31]. The positive association between the density of garbage accumulation points and *Ae. aegypti* egg counts further highlights the role of the built environment in shaping vector ecology. Areas with persistent waste accumulation tend to concentrate discarded containers, irregular surfaces, and shaded microhabitats capable of retaining rainwater, thereby expanding the availability of breeding sites [32,33]. Furthermore, in southern Thailand, the presence of waste piles near homes increased the risk of asymptomatic CHIKV infection by 2.23 times and symptomatic CHIKV infection by 3.97 times [34]. These findings highlight the importance of maintaining regular garbage collection, proper disposal of accumulated materials, and community awareness of waste management as supportive components of vector control, while recognizing that, on their own, such measures are unlikely to produce consistent reductions in CHIKV transmission risk.

Our study also demonstrated an association between advanced age and a higher prevalence of CHIKV, suggesting that older adults are particularly vulnerable. This finding aligns with previous studies reporting a relationship between older age and increased prevalence [22,23,35]. In the Brazilian context, where widespread transmission began only in the last decade, this association is unlikely to reflect cumulative lifetime exposure alone. Instead, it may be partially explained by behavioral and social factors, such as spending more time within or around the household environment, where vector density is highest, as well as by age-related immunological differences that may influence susceptibility or seroconversion. Together, these mechanisms highlight older adults as a priority group for prevention and risk communication in vulnerable urban communities.

Structural characteristics of residences, such as unplastered brick walls, were also identified as risk factors for CHIKV exposure in our study. In Salvador, Anjos *et al*. (2020) reported similar associations with unplastered brick walls, while also showing that neighborhood infrastructure, as unpaved streets, were linked to prior CHIKV infection at the neighborhood scale in Salvador [36]. In a multi community-level analysis, Travis *et al.* (2025) found that environmental deficiencies and topographical features, including hillside location, elevation gradients, and proximity to surface water, were associated with higher CHIKV seroprevalence [37], indicating that micro-environmental conditions shape infection patterns beyond household structure alone. Our study extends these findings by integrating serological, entomological, and environmental data within a single informal settlement. This finer spatial resolution reveals that the same structural vulnerabilities identified at broader scales also generate pronounced heterogeneity in exposure over distances of only ten meters, linking neighborhood-level disadvantage to household-level transmission dynamic. These features are not merely markers of poverty, but active components of the domestic environment that shape mosquito ecology. Such conditions may facilitate water accumulation and create breeding sites for *Ae. aegypti*, the vector of the virus. In addition, the unpaved yards may be associated with more vegetated and humid environments which could be more attractive to female *Aedes*, given its preference to oviposit close to the ground and water containers with organic compounds [38].

The positive trends observed between the risk of exposure to chikungunya and the predicted *Ae. aegypti* egg count, as well as the Land Surface Temperature, are relevant despite not being statistically significant and warrant further investigation. Our decision to retain these variables was not based solely on their established role in the vector-borne disease literature. Rather, it was motivated by both their biological relevance and the substantial spatial heterogeneity observed within the study area. Despite the relatively fine spatial scale of the analysis, land surface temperature varied by approximately 5°C across the community (ranging from 28°C to 33°C), while predicted spatial egg counts varied more than threefold (approximately 25–127 eggs). Likewise, predicted seroprevalence showed marked spatial heterogeneity, ranging from approximately 15% to 74% across the study area. These patterns suggest that meaningful environmental and entomological gradients exist even within this relatively small urban setting. In the context of our study area, this suggests that local microclimatic conditions may play a critical role in modulating vector abundance and transmission dynamics.

Our study, despite relying on ovitraps, which capture only oviposition activity rather than the adult mosquito population (the transmission stage), suggests that fine scale ovitraps data can contribute as one layer within an integrated risk framework. Rather than serving as a standalone indicator of chikungunya transmission, entomological surfaces should be interpreted in conjunction with serological and epidemiological data to identify areas of increased risk of chikungunya infection.

The relationship between temperature and chikungunya incidence has been previously described in Salvador [21], aligning with both vector biology and viral replication dynamics observed in experimental studies [39]. In our study area, land surface temperature exhibited substantial fine-scale spatial variation, with some locations reaching temperatures up to five degrees higher than others (Fig 1d). Land surface temperature maps of Salvador city, showed that hotter neighborhoods frequently reached 30–32°C, a range close to the thermal optimum for *Aedes aegypti* development and chikungunya virus transmission [40–44]. These temperature ranges are consistent with experimental and modeling studies showing favorable conditions for both vector development and chikungunya viral transmission [45,46]. Similar intra-urban thermal gradients have been reported in other tropical and subtropical cities and associated with spatial heterogeneity in arboviral transmission risk [26,40–42]. Our findings therefore support the need to interpret serological exposure and entomological indicators within a fine-scale thermal framework that accounts for urban heat islands. Moreover, projected increases in urbanization and temperature associated with climate change may further exacerbate urban heat island effects, potentially expanding areas with environmental conditions favorable for mosquito proliferation and arboviral transmission, particularly in densely populated and socio-environmentally vulnerable urban communities [47]. Adaptation measures aimed at reducing intra-urban temperature disparities and mitigating urban heat islands, such as increasing urban vegetation and tree canopy cover, implementing green and reflective roofs, expanding permeable surfaces, and improving access to shaded public spaces, could reduce local heat exposure and, in turn, lessen the impact of mosquito-borne diseases in these high-risk areas [44]. Integrating these adaptation strategies into urban planning may help modify microclimatic conditions that favor vector persistence and transmission, particularly in socio-environmentally vulnerable neighborhoods.

This study has several limitations that should be considered. First, climatic conditions and vector abundance vary over time, and our analyses capture a specific temporal window within a dynamic transmission system. Vector sampling was conducted over a single one-month period, providing only a short temporal snapshot of oviposition activity. Given that *Ae. aegypti* populations respond rapidly to rainfall and temperature fluctuations, this limited window may not capture the full seasonal variability of vector abundance. Incorporating longitudinal entomological and climatic data would allow a more explicit characterization of seasonal and interannual variability in future work. Second, the study was conducted within a single vulnerable community in Salvador, which may limit direct generalization to other urban contexts. However, the objective was not to produce citywide estimates, but rather to interrogate fine-scale processes operating within informal settlements. In this sense, the analytical framework and mechanisms identified here are likely transferable to similar socio-environmental settings. In addition, *Ae. aegypti* abundance was assessed using ovitraps, which measure oviposition activity rather than adult mosquito density, the stage directly responsible for transmission. Nevertheless, ovitraps are highly sensitive, operationally feasible, and widely validated as proxies of spatial vector distribution [48,49] making them well suited for fine-scale risk mapping in resource-constrained environments. Regarding CHIKV exposure analyses, our study had a cross-sectional design; therefore, we were unable to link seroconversion to a specific time period. In addition, exposure may have occurred outside the study area, potentially attenuating or obscuring the environmental associations evaluated. Despite these limitations, the observed associations are consistent with prior studies and provide meaningful insights into the spatial patterns of CHIKV exposure in vulnerable urban settings. Future studies specifically designed to evaluate chikungunya incidence, with tighter temporal alignment between entomological surveillance and epidemiological outcomes and reduced influence of external control interventions, are needed to better assess the utility of fine-scale *Ae. aegypti* egg count metrics for identifying areas at higher transmission risk.

Our findings demonstrate that chikungunya transmission in vulnerable urban communities is shaped by fine-scale socio-environmental heterogeneity that simultaneously structures vector ecology and human exposure. Rather than being uniformly distributed, risk emerges from localized interactions between housing conditions, environmental degradation, microclimate, and household-level vulnerability. High-resolution spatial estimates of *Ae. aegypti* abundance, when integrated with serological and socio-environmental data, provide a powerful framework for identifying micro-hotspots of transmission. While many recommended actions, such as yard cleaning and elimination of breeding sites, are well established, our contribution lies in redefining how these measures are spatially deployed. Rather than uniform coverage, our results support a precision public health approach, in which interventions are concentrated in micro-hotspots identified through the integration of entomological, environmental, and social data. Embracing this spatial complexity allows public health actions to move from homogeneous control toward context-specific strategies capable of reducing arboviral burden and advancing environmental justice in high-risk settings.

## Methods

### Ethics statement

This study was approved by the Research Ethics Committee of the Institute of Collective Health/Federal University of Bahia (CEP/ISC/UFBA) and by the National Commission for Ethics in Research (CONEP) linked to the Brazilian Ministry of Health under protocol number CAAE n° 35405320.0.1001.5030, approval opinion number 4.510.173/2021. Written informed consent was obtained from all participants or their legal representatives prior to their inclusion in the study.

### Study site and population

A cross-sectional study was conducted in the Marechal Rondon community (12° 54’ 44.622” S, 38° 28’ 23.024” W), located in the periphery of Salvador, Bahia, Brazil. Salvador is the capital of Bahia state and the fourth largest Brazilian city with 2.4 million persons and an extension of 693km^2^, is situated northeast Brazil (38°26’09"W, 12°55’32"S). The climate is tropical, with little temperature variation throughout the year, and a daily mean temperature of 25.2 °C. The overall mean average annual precipitation is 1,781 mm, characterized by seasonal variability, with the driest season occurring in summer and the wettest season in autumn [50]. In particular, the Marechal Rondon neighborhood faces substantial challenges related to socioeconomic vulnerability, deficient infrastructure, and inadequate sanitation. These conditions include open sewage channels, unpaved streets, accumulation of solid waste, and areas of stagnant water [34,35], all of which contribute to an elevated risk of infectious diseases [36,37]. Within this neighborhood, we delineated the study area to include households located within 30 m of open sewage channels or a lake, representing zones of heightened socio-environmental vulnerability (Fig 1). The study area spanned 0.31 km^2^ and included a total of 1,215 households. All households were geocoded and mapped. During the study conducted between October 2021 and June 2022, we invited all residents by house-by-house visits within the study area who met the inclusion criteria: aged ≥5 years and slept in the study area for at least 3 nights per week, to participate in the research. Those who agreed provided written informed consent if they were adults. For minors, written informed consent was obtained from a parent or legal guardian, alongside written informed assent from the participant. All enrolled participants who provided consent or assent subsequently underwent peripheral blood collection, and blood samples were obtained from all participants for serological analyses according to the study protocol.

### Data collection

The questionnaires applied addressed a range of socioeconomic, demographic, structural, and environmental aspects. They included information on sex, age, head of household education, self-reported skin color, employment status (formal or informal), head of household income, number of residents in the household, number of rooms, type of street paving, material of the dwelling walls (concrete, uncovered brick, or wood), access to piped water, occurrence of water shortages nearby, water storage practices, presence of a yard, history of flooding, garbage collection frequency, and observation of mosquitoes in the last seven days. This recall period was chosen to capture recent mosquito presence while minimizing recall bias and reflecting short-term environmental and climatic conditions that influence mosquito activity. Additionally, a proxy measure of socio-economic vulnerability defined food insecurity based on the self-reported occurrence of insufficient financial resources to purchase food within the previous six months.

This information was collected using standardized questionnaires through the REDCap platform. Additionally, participants provided a blood sample for serological testing.

### Geospatial assessment

We used three geographical and satellite derived environmental variables. A World View image (0.3m resolution, date: April 2022) with green, blue, red and near infrared bands was classified by supervised classification using Erdas Imagine Software [51] into four land cover classes: impervious surface, exposed soil, water and vegetation. Then, we extracted the percentage of land cover classes around a buffer of 50 m around each household with the landscape metrics package in R [52,53].

Mean land surface temperature (LST) spatial pattern was obtained from the Landsat 8 images that presented a cloud coverage of less than 10% available close to the studied period. (Dates: March 13, 2016; October 15, 2016; January 6, 2018; May 14, 2018; August 2, 2018; October 6, 2018; December 8, 2018; January 25, 2019; and December 11, 2019). The availability of cloud-free Landsat scenes over the study area was limited; therefore, the temporal window was extended back to 2016 to ensure an adequate number of images meeting the cloud coverage criterion. Then the mean LST was obtained for each household location.

A proxy of flood susceptibility was obtained from a Topographical Wetness Index Map (TWI), which followed the methodology described by Beven (1997) [54]. The TWI is derived from a mechanistic hydrological model that considers the slope of each cell and its water catchment area. The TWI map used in this study was originally produced for the city of Salvador by Pedra [55] and has a resolution of 5m. Given the nature of this index, and the observation of large variation over small distances, we opted to extract the mean and maximum TWI in a buffer of 30m around each household.

Elevation was obtained from the Digital Elevation Model (5m resolution) available from the Salvador Municipality geoservice [56].

In September 2022, we conducted a field survey to assess environmental vulnerabilities across the entire public area. During this survey, vulnerabilities such as the accumulation of trash, garbage, and building materials, as well as broken storm drains and open sewages, were identified and geolocated. The criteri on for defining the accumulation of trash, garbage, and building materials was that these materials covered more than 50% of a 1-square-meter area.

Subsequently, a kernel density map was created for each type of vulnerability, as well as for all elements combined, using a bandwidth of 50 meters and Epanechnikov kernel function [57]. This resulted in raster layers representing the density of environmental vulnerabilities, from which each element and all joined vulnerability density values were extracted for each household location. Also, the distance to the closest open sewage was estimated for each household location.

### Laboratory methods

The collected blood samples were refrigerated at 2–8°C and transported to the Pathology and Molecular Biology Laboratory at the Gonçalo Moniz Institute, Fiocruz, Bahia. Serum was then separated by centrifugation, aliquoted, and stored at -20°C until further analysis.

The IgG antibodies against chikungunya were analyzed using the IgG ELISA kit of EUROIMMUN (Lubeck, Germany), following the manufacturer's guidelines. Results were expressed semi-quantitatively by calculating a ratio, obtained by dividing the absorbance (OD) of a sample by the OD of a calibrator serum included in the same assay. Index values <0.8 were considered negative, values between 0.8 to <1.1 were indeterminate, and values ≥1.1 were considered positive. We repeated the tests on samples that showed indeterminate results in the initial test and considered the obtained results as definitive.

### *Aedes aegypti* spatial distribution

To assess the spatial distribution of *Ae. aegypti*, we conducted a survey using ovitraps, which consisted of a black plastic container with 300 mL of water and a 4 × 10 cm wooden paddle. The sampling design was based on the construction of a grid of hexagons, each with a side length of 30 meters, covering the entire study area (S1 Fig). This size was chosen based on the ability to identify *Ae. aegypti* clusters around breeding sites. In May 2022, one household participating in the serological survey was selected within each hexagon. Two ovitraps were installed, one in the intradomicile and the other in the peridomestic area, at each selected household and remained in place for five to seven days. After this period, the ovitraps were collected, and the *Aedes* spp. eggs were identified and counted under a stereoscopic microscope using standard morphological criteria [30], with *Ae. aegypti* representing the established dominant vector in the study area [58]. A negative binomial geostatistical model was constructed to generate a smoothed estimate of the spatial distribution of ovitrap egg counts, serving as a proxy for *Ae. aegypti* abundance [48], across the study area. This was done by applying a predictive INLA-SPDE model without covariates with the outcome of ovitrap egg count in each measured location. The resulting model allowed us to create a raster layer representing the spatial distribution of *Ae. aegypti* abundance, from which we extracted estimated values at the participants’ home locations.

### Data analysis

To estimate the association between sociodemographic, environmental, and spatial patterns of *Ae. aegypti* abundance and human exposure to CHIKV, we developed an integrated modeling framework that explicitly accounted for these multiple dimensions. We fitted a geostatistical model using the Integrated Nested Laplace Approximation (INLA) with the Stochastic Partial Differential Equation (SPDE) approach [59,60].

As an initial step, we conducted descriptive and exploratory analyses separately for each outcome. We fitted bivariate non-spatial generalized linear mixed models (GLMMs), specifying a zero-inflated negative binomial distribution for the *Ae. aegypti* outcome and a binomial distribution for the human exposure outcome. Outcomes were defined as (i) *Ae. aegypti* egg counts recorded in ovitraps and (ii) individual serological status for CHIKV exposure. Sociodemographic and environmental variables were included as fixed effects (see S3 Table), and a random intercept at the household level was specified to account for within-household clustering. All continuous covariates were standardized prior to model fitting using the *scale* function in R, which centers each variable at its mean and rescales it by its standard deviation. For the *Ae. aegypti* egg count model, the appropriateness of the distributional assumption and the presence of overdispersion were evaluated using the testDispersion function from the *performance* package [61]. To account for slight variations in exposure duration, the natural logarithm of number of days each ovitrap remained active was incorporated as an offset in the statistical models (see S1 Table).

Subsequently, multivariable GLMMs including all candidate predictors were fitted for each outcome in order to test multicollinearity among predictors, which was assessed using the variance inflation factor (VIF), and variables with VIF values greater than 4 were excluded.

To explicitly account for spatial autocorrelation, the multivariable models were subsequently refitted within a Bayesian geostatistical framework using the INLA-SPDE approach, as implemented in the INLA package. Spatial dependence was modeled using a Matérn covariance function parameterized through the SPDE formulation. Penalized complexity (PC) priors were specified for the spatial random field using the pc.matern parameterization, with the smoothness parameter fixed at α = 2, corresponding to a twice-differentiable Gaussian random field. This specification imposes a realistic degree of spatial smoothness while allowing the spatial structure to be flexibly informed by the data. The PC prior penalizes deviations from a base model with no spatial variation, thereby regularizing estimation of the spatial range (ρ) and marginal variance (σ^2^) and reducing the risk of overfitting.

A manual backward-selection procedure was applied, guided by the Watanabe–Akaike information criterion (WAIC), to derive final spatial models representing an optimal trade-off between explanatory power, parsimony, and biological plausibility. Biological plausibility informed the retention of candidate variables, but model selection did not preferentially favor models because estimated coefficients conformed to a priori expectations regarding effect direction.

In addition, to visualize the spatial pattern of chikungunya exposure, we fitted a Bayesian geostatistical model with a binomial likelihood and a logit link within the same INLA-SPDE framework described above, excluding covariates. This model was used for descriptive purposes to estimate a continuous spatial surface representing the underlying seroprevalence across the study area.

## Supporting information

S1 Fig Sampling grid illustrating surveyed households and mosquito ovitrap locations within each hexagonal polygon across the study area. The high-resolution base orthoimage used for map visualization was obtained from the Salvador Municipality cartographic services via its WMS service available at https://www.sefaz.salvador.ba.gov.br/geosalvador/home/item.html?id=139b22a13a654f779bbbbd8cf350347d (accessed February 2026). The WMS is provided as publicly accessible municipal open data for consultation and reuse, subject to source attribution in accordance with the municipality’s open data and public information policies (https://www.sefaz.salvador.ba.gov.br/geosalvador/home/item.html?id=a455c391abc4495b9ce96693c4f288b6).
(TIF)

S1 Table Ranking of candidate models with ΔWAIC < 2 for the outcome of *Aedes aegypti* egg counts in ovitraps deployed in the Marechal Rondon community. Models are ordered from lowest to highest WAIC, indicating comparable support among the best-performing model structures for explaining variation in oviposition counts.
(DOCX)

S2 Table Ranking of candidate models with ΔWAIC < 2 for the chikungunya virus serostatus outcome in the Marechal Rondon community. Models are ordered from lowest to highest WAIC, representing the set of best-supported model specifications with comparable explanatory power for variation in serological status.
(DOCX)

S3 Table Definitions and descriptions of sociodemographic and environmental predictor variables included in the models for each outcome. The table details variable nomenclature, operational definitions, measurement scales, and the specific outcomes (e.g., *Aedes aegypti* oviposition counts and chikungunya virus serostatus) for which each predictor was considered.
(XLSX)

S4 Table Full list of legends for the Supporting Information data bases files (S1 Data and S2 Data). (DOCX)

S1 Data Full database of ovitrap surveillance data used in the study. (XLSX)

S2 Data Full database of chikungunya virus data used in the study. (CSV)
