# Supplementary material for: Socio-environmental assessment of Aedes aegypti distribution and risk of chikungunya in vulnerable community
Source: PLoS Negl Trop Dis. 2026 Oct 12;20(10):e0014776. doi: 10.1371/journal.pntd.0014776 (PMC13712677; doi:10.1371/journal.pntd.0014776)
Supplement: Supplementary file 1 [file pntd.0014776.s013.pdf]

July 23, 2026
Editorial Board
PLOS Neglected Tropical Diseases
Re: Response to Reviewers
Manuscript Title: *Socio-environmental assessment of Aedes aegypti distribution and risk of chikungunya in a vulnerable community*

Dear Editors and Reviewers,

We appreciate the editors´ and reviewers' thoughtful and constructive feedback, and we are grateful for their recognition of the value of our work.

As requested, we have responded to each comment point by point. To facilitate review, our responses were provided in blue italic font, clearly distinguishing them from the original reviewer comments.

Sincerely,

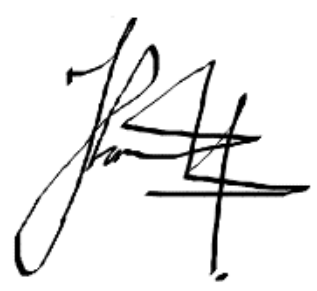

Hussein Khalil, PhD, on behalf of the authors

Editor´s comments:

Indicate what the items in bold mean as a footnote for each table. Please use the 'track changes' feature to indicate any changes made in the revised documents rather than highlighting the sections. Please redo the revision (in addition to the ones suggested here) using this 'track changes' feature to allows for an accurate assessement.

We have added a footnote to each table indicating that values shown in bold represent statistically significant results ($p < 0.05$) for frequentist analyses or estimates with 95% credible intervals that do not include 1 for Bayesian models. In addition, we have revised the manuscript using the Track Changes feature, and all modifications—including those made in response to both the current and previous round of review—are now displayed using track changes, as requested.

Revise the response to reviewer 2 on WAIC/model-selection explanation. The phrase "effects ... consistent with current biological understanding" could be interpreted as selecting a model partly because the direction of the result conformed to expectations. This premise risks appearing post hoc. Make it clear in the MS that biological plausibility informed candidate model specification or interpretation, whereas final selection considered predictive fit and parsimony without preferentially selecting models because coefficients had expected directions.

We have revised the answer of the WAIC-based model selection procedure to clarify the role of biological plausibility:

> *Models with ΔWAIC < 2 were considered to have comparable predictive performance; however, final model selection was guided by both statistical performance and biological interpretability. When competing models exhibited similar WAIC values, preference was given to the more parsimonious model, provided that retained covariates were biologically plausible and supported an ecologically meaningful interpretation of the system. The direction of estimated coefficients was not used as a criterion for model selection.*

We also revised the corresponding statement in the Methods section, which now reads:

> *A manual backward-selection procedure was applied, guided by the Watanabe–Akaike information criterion (WAIC), to derive final spatial models representing an optimal trade-off between explanatory power, parsimony, and biological plausibility. Biological plausibility informed the retention of candidate variables, but model selection did not preferentially favor models because estimated coefficients conformed to a priori expectations regarding effect direction.*

With regards to the supplementary material, please revise the Data Availability Statement to clarify that exact household coordinates have been withheld to protect participant confidentiality and, where these data are necessary for reproducibility, provide details of the appropriate institutional mechanism through which qualified researchers may request controlled access in accordance with ethical and data-protection requirements.

We have revised the Data Availability Statement to clarify that exact household coordinates have been withheld to protect participant confidentiality. We also added information on the institutional mechanism through which qualified researchers may request controlled access to these data in accordance with ethical and data-protection requirements.

The revised statement now reads:

> *Exact household coordinates have been withheld to protect participant confidentiality. When these data are necessary for reproducibility, controlled access may be granted upon reasonable request, subject to adequate justification and ethics committee approval. Requests should be directed to the corresponding author (hussein.khalil@slu.se). To obtain access, researchers must submit a research proposal, evidence of ethical approval, and a data management plan to the Research Ethics Committee of the Gonçalo Moniz Institute - Fiocruz/BA (cep.igm@fiocruz.br). If approved, an anonymized dataset containing the information required for the proposed analyses will be provided.*

Figure resubmission: While revising your submission, we strongly recommend that you use PLOS's NAAS tool (https://ngplosjournals.pagemajik.ai/artanalysis) to test your figure

files. NAAS can convert your figure files to the TIFF file type and meet basic requirements (such as print size, resolution), or provide you with a report on issues that do not meet our requirements and that NAAS cannot fix.

After uploading your figures to PLOS's NAAS tool - https://ngplosjournals.pagemajik.ai/artanalysis, NAAS will process the files provided and display the results in the "Uploaded Files" section of the page as the processing is complete. If the uploaded figures meet our requirements (or NAAS is able to fix the files to meet our requirements), the figure will be marked as "fixed" above. If NAAS is unable to fix the files, a red "failed" label will appear above. When NAAS has confirmed that the figure files meet our requirements, please download the file via the download option, and include these NAAS processed figure files when submitting your revised manuscript.

We used the PLOS NAAS tool to assess and process all figure files. Where necessary, the tool corrected the files to meet the journal's technical requirements, and the resulting NAAS-processed figure files have been included with the revised manuscript.

Reproducibility: To enhance the reproducibility of your results, we recommend that authors of applicable studies deposit laboratory protocols in protocols.io, where a protocol can be assigned its own identifier (DOI) such that it can be cited independently in the future. Additionally, PLOS ONE offers an option to publish peer-reviewed clinical study protocols. Read more information on sharing protocols at https://plos.org/protocols?utm_medium=editorial-email&utm_source=authorletters&utm_campaign=protocols

The laboratory methods used in this study followed the manufacturers' recommended protocols for the commercial assays employed, which are cited in the manuscript. Since no original or modified laboratory protocol was developed, we did not deposit a protocol in protocols.io.
