# Supplementary material for: Socio-environmental assessment of Aedes aegypti distribution and risk of chikungunya in vulnerable community
Source: PLoS Negl Trop Dis. 2026 Oct 12;20(10):e0014776. doi: 10.1371/journal.pntd.0014776 (PMC13712677; doi:10.1371/journal.pntd.0014776)
Supplement: Supplementary file 2 [file pntd.0014776.s002.docx]

|  | Model set | WAIC | ΔWAIC |
| --- | --- | --- | --- |
| 1 | Location + Garbage points density + Food insecurity + Type of yard + offset(log(ovitrap exposure days)) | 1486.55 | 0.00 |
| 2 | Location + Garbage points density + Food insecurity + Open sewage nearby + Type of yard + Head of household income + offset(log(ovitrap exposure days)) | 1487.51 | 0.96 |
| 3 | Location + Garbage points density + Food insecurity + Type of yard + Head of household income + offset(log(ovitrap exposure days)) | 1487.59 | 1.04 |
| 4 | Location + Garbage points density + Food insecurity + Open sewage nearby + Type of yard + offset(log(ovitrap exposure days)) | 1487.77 | 1.22 |
