# Supplementary material for: Socio-environmental assessment of Aedes aegypti distribution and risk of chikungunya in vulnerable community
Source: PLoS Negl Trop Dis. 2026 Oct 12;20(10):e0014776. doi: 10.1371/journal.pntd.0014776 (PMC13712677; doi:10.1371/journal.pntd.0014776)
Supplement: Supplementary file 3 [file pntd.0014776.s003.docx]

|  | Model set | WAIC | ∆WAIC |
| --- | --- | --- | --- |
| 1 | Age + Wall material + Type of yard + Mosquito presence self-report + Water storage + Land Surface Temperature + Egg count prediction | 920.67 | 0 |
| 2 | Age + Wall material + Type of yard + Mosquito presence self-report + Water storage + Storm drain density + Land Surface Temperature + Egg count prediction + Vegetation class coverage | 920.75 | 0.08 |
| 3 | Age + Pipe water service + Wall material + Type of yard + Mosquito presence self-report + Water storage + + Land Surface Temperature + Egg count prediction + Vegetation class coverage | 920.76 | 0.09 |
| 4 | Age + Wall material + Type of yard + Mosquito presence self-report + Water storage + Storm drain density + Land Surface Temperature + Egg count prediction | 920.8 | 0.13 |
| 5 | Age + Wall material + Type of yard + Mosquito presence self-report + Water storage + Land Surface Temperature + Egg count prediction + Vegetation class coverage | 921.02 | 0.35 |
| 6 | Age + Wall material + Type of yard + Mosquito presence self-report + Water storage + Land Surface Temperature + Egg count prediction + Vegetation class coverage | 921.02 | 0.35 |
| 7 | Age + Head of Household schooling + Wall material + Type of yard + Mosquito presence self-report + Water storage + Storm drain density + Land Surface Temperature + Egg count prediction | 921.04 | 0.37 |
| 8 | Head of Household schooling + Wall material + Type of yard + Mosquito presence self-report + Water storage + Storm drain density + Land Surface Temperature + Egg count prediction + Vegetation class coverage | 921.06 | 0.39 |
| 9 | Age + Head of Household schooling + Wall material + Type of yard + Mosquito presence self-report + Water storage + Storm drain density + Land Surface Temperature + Egg count prediction + Vegetation class coverage | 921.07 | 0.4 |
| 10 | Age + Head of Household schooling + Pipe water service + Wall material + Type of yard + Mosquito presence self-report + Water storage + Storm drain density + Land Surface Temperature + Egg count prediction + Vegetation class coverage | 921.08 | 0.41 |
| 11 | Age + Head of Household schooling + Wall material + Type of yard + Mosquito presence self-report + Water storage + Land Surface Temperature + Egg count prediction + Vegetation class coverage | 921.26 | 0.59 |
| 12 | Age + Wall material + Type of yard + Mosquito presence self-report + Water storage + Storm drain density + Land Surface Temperature + Egg count prediction + Impervious surface class coverage | 921.27 | 0.6 |
| 13 | Age + Type of yard + Mosquito presence self-report + Water storage + Storm drain density + Land Surface Temperature + Egg count prediction + Vegetation class coverage | 921.36 | 0.69 |
| 14 | Age + Wall material + Type of yard + Mosquito presence self-report + Water storage + Land Surface Temperature | 921.5 | 0.83 |
| 15 | Age + Head of Household schooling + Wall material + Type of yard + Mosquito presence self-report + Water storage + Storm drain density + Land Surface Temperature + Egg count prediction + Impervious surface class coverage | 921.54 | 0.87 |
| 16 | Age + Head of Household schooling + Type of yard + Mosquito presence self-report + Water storage + Storm drain density + Land Surface Temperature + Egg count prediction + Vegetation class coverage | 921.6 | 0.93 |
| 17 | Age + Wall material + Type of yard + Mosquito presence self-report + Water storage + Storm drain density + Land Surface Temperature + Vegetation class coverage | 921.62 | 0.95 |
| 18 | Age + Wall material + Type of yard + Mosquito presence self-report + Water storage + Storm drain density + Trash points density + Land Surface Temperature + Egg count prediction + Vegetation class coverage | 921.69 | 1.02 |
| 19 | Age + Wall material + Type of yard + Mosquito presence self-report + Water storage + Egg count prediction | 921.74 | 1.07 |
| 20 | Age + Wall material + Type of yard + Mosquito presence self-report + Water storage + Storm drain density + Land Surface Temperature | 921.74 | 1.07 |
| 21 | Age + Wall material + Type of yard + Mosquito presence self-report + Water storage + Storm drain density + Land Surface Temperature | 921.75 | 1.08 |
| 22 | Age + Head of Household schooling + Wall material + Type of yard + Mosquito presence self-report + Water storage + Storm drain density + Trash points density + Land Surface Temperature + Egg count prediction | 921.84 | 1.17 |
| 23 | Age + Wall material + Type of yard + Mosquito presence self-report + Water storage + Land Surface Temperature + Vegetation class coverage | 921.84 | 1.17 |
| 24 | Head of Household schooling + Wall material + Type of yard + Mosquito presence self-report + Water storage + Storm drain density + Trash points density + Land Surface Temperature + Egg count prediction + Vegetation class coverage | 921.91 | 1.24 |
| 25 | Age + Wall material + Type of yard + Mosquito presence self-report + Water storage + Storm drain density + Egg count prediction + Vegetation class coverage | 921.95 | 1.28 |
| 26 | Age + Head of Household schooling + Pipe water service + Wall material + Type of yard + Mosquito presence self-report + Water storage + Storm drain density + Trash points density + Land Surface Temperature + Egg count prediction + Vegetation class coverage | 921.97 | 1.3 |
| 27 | Age + Head of Household schooling + Wall material + Type of yard + Mosquito presence self-report + Water storage + Storm drain density + Trash points density + Land Surface Temperature + Egg count prediction + Vegetation class coverage | 921.97 | 1.3 |
| 28 | Age + Head of Household schooling + Wall material + Type of yard + Mosquito presence self-report + Water storage + Storm drain density + Land Surface Temperature + Vegetation class coverage | 922 | 1.33 |
| 29 | Age + Head of Household schooling + Wall material + Type of yard + Mosquito presence self-report + Water storage + Building materials points density + Storm drain density + Land Surface Temperature + Egg count prediction + Vegetation class coverage | 922.14 | 1.47 |
| 30 | Age + Head of Household schooling + Wall material + Type of yard + Mosquito presence self-report + Water storage + Trash points density + Land Surface Temperature + Egg count prediction + Vegetation class coverage | 922.14 | 1.47 |
| 31 | Wall material + Type of yard + Mosquito presence self-report + Water storage + Storm drain density + Land Surface Temperature + Egg count prediction + Vegetation class coverage | 922.26 | 1.59 |
| 32 | Age + Head of Household schooling + Wall material + Type of yard + Mosquito presence self-report + Water storage + Storm drain density + Egg count prediction + Vegetation class coverage | 922.3 | 1.63 |
| 33 | Wall material + Type of yard + Mosquito presence self-report + Water storage + Storm drain density + Land Surface Temperature + Egg count prediction + Vegetation class coverage | 922.42 | 1.75 |
| 34 | Age + Wall material + Type of yard + Mosquito presence self-report + Water storage + Building materials points density + Storm drain density + Trash points density + Land Surface Temperature + Egg count prediction + Vegetation class coverage | 922.5 | 1.83 |
| 35 | Age + Head of Household schooling + Wall material + Type of yard + Mosquito presence self-report + Water storage + Storm drain density + Trash points density + Land Surface Temperature + Egg count prediction + Impervious surface class coverage | 922.51 | 1.84 |
| 36 | Age + Head of Household schooling + Type of yard + Mosquito presence self-report + Water storage + Storm drain density + Trash points density + Land Surface Temperature + Egg count prediction + Vegetation class coverage | 922.66 | 1.99 |
