# Supplementary material for: Socio-environmental assessment of Aedes aegypti distribution and risk of chikungunya in vulnerable community
Source: PLoS Negl Trop Dis. 2026 Oct 12;20(10):e0014776. doi: 10.1371/journal.pntd.0014776 (PMC13712677; doi:10.1371/journal.pntd.0014776)
Supplement: Supplementary file 4 [file pntd.0014776.s005.docx]

**S4 Table:** Full list of legends for the Supporting Information data bases files ("S1 Data and "S2 Data")

| File | Variable | Description (Variable units or categories) |
| --- | --- | --- |
| S1 Data‍ | id_ovitret | Unique ovitrap identification number |
| ‍ | id_Proj | Unique location identification number |
| ‍ | casanova | Identification of the hosehold were ovitrap was placed |
| ‍ | eggs | Number of Aedes aegypti eggs counted on the ovitrap wooden paddle. |
| ‍ | X | Longitude coordinate of participant household/location (EPSG: 31984).* |
| ‍ | Y | Latitude coordinate of participant household/location (EPSG: 31984).* |
| ‍ | HH_Income | Houshold income |
| ‍ | Res_room | Number of residents per room (household crowding indicator) |
| ‍ | fi_screen2 | Food vulnerability (Yes/no) |
| ‍ | Pipe_water | Access to piped water supply (Yes/no) |
| ‍ | water_storage | Presence of water storage containers in household |
| ‍ | wall_material | Predominant household wall construction material (Plasted wall/Uncovered brick or other than masonry) |
| ‍ | Yard_cover | Type of yard surface cover surrounding household (Unpaved yard/paved yard/not yard) |
| ‍ | Location | Location were ovitrap was placed (Intra/Peridomicile) |
|  | Days | Number of days that ovitrap remained active |
| ‍ | open_sewage | Presence of open sewage nearby the household in a radius of 20 m (Yes/no) |
| ‍ | Dom_waste | Location where household residents dispose of their garbage. |
| ‍ | Waste_frequency | Frequency of garbage collection service in the street (Daily/not daily) |
| ‍ | Altitud | Elevation above sea level (m) |
| ‍ | LST_SSA | Land Surface Temperature (°C) derived from remote sensing |
| ‍ | Storm_drains | Proximity of storm drains around 20 mts of household (Yes/no) |
| ‍ | TWI_max | Maximum Topographic Wetness Index value around household in a buffer of 20 mts |
| ‍ | Trash | Kernel density of accumulated trash points in the household location |
| ‍ | Garbage | Kernel density of accumulated garbage points in the household location |
| ‍ | Const_Material | Kernel density of accumulated construction materials points in the household location |
| ‍ | pland_1_50 | Percentage of impervious landcover class within a 50-m buffer (PLAND metric) |
| ‍ | pland_4_50 | Percentage of vegetation landcover class within a 50-m buffer (PLAND metric) |
| ‍ | pland_3_50 | Percentage of baresoil landcover class within a 50-m buffer (PLAND metric) |
| ‍ | pland_2_50 | Percentage of water landcover class within a 50-m buffer (PLAND metric) |
| S2 Data | idnova | Unique participant identification number |
|  | X* | Longitude coordinate of participant household/location (EPSG: 31984) |
|  | Y* | Latitude coordinate of participant household/location (EPSG: 31984) |
|  | casanova | Household identification code |
|  | Chikv_serostatus | Chikungunya virus serological status (positive/negative) |
|  | Sex | Participant sex (Male/Female) |
|  | Age | Participant age (years) |
|  | Race | Self-reported skin color (black or mixed-race/White/Others) |
|  | Marital | Marital status (Single/Stable union/legally married/Separated, diverced or widowed) |
|  | Schooling | Completed schooling years |
|  | Income | Individual income |
|  | Res_room | Number of residents per room (household crowding indicator) |
|  | fi_screen2 | Food vulnerability (Yes/no) |
|  | Pipe_water | Access to piped water supply (Yes/no) |
|  | water_storage | Presence of water storage containers in household |
|  | wall_material | Predominant household wall construction material (Plasted wall/Uncovered brick or other than masonry) |
|  | Yard_cover | Type of yard surface cover surrounding household (Unpaved yard/paved yard/not yard) |
|  | mosquito | Presence or abundance of mosquitoes observed at household (Yes/no) |
|  | Altitud | Elevation above sea level (m) |
|  | LST_SSA | Land Surface Temperature (°C) derived from remote sensing |
|  | Storm_drain | Kernel density of storm drain points in the household location |
|  | TWI_max | Maximum Topographic Wetness Index value around household in a buffer of 20 mts |
|  | Trash | Kernel density of accumulated trash points in the household location |
|  | Garbage | Kernel density of accumulated garbage points in the household location |
|  | Const_Material | Kernel density of accumulated construction materials points in the household location |
|  | Pland_1_50 | Percentage of impervious landcover class within a 50-m buffer (PLAND metric) |
|  | Pland_4_50 | Percentage of vegetation landcover class within a 50-m buffer (PLAND metric) |
|  | Pland_3_50 | Percentage of baresoil landcover class within a 50-m buffer (PLAND metric) |
|  | Pland_2_50 | Percentage of water landcover class within a 50-m buffer (PLAND metric) |
|  | ov_MR_zinb1 | Spatial predicted Ae. aegypti egg count |
| *Household geographic coordinates were removed from the publicly available dataset to protect participant confidentiality. Access to precise spatial data may be granted upon reasonable request, subject to ethical approval and data-sharing agreements. | | |
