# Supplementary material for: Socio-environmental assessment of Aedes aegypti distribution and risk of chikungunya in vulnerable community
Source: PLoS Negl Trop Dis. 2026 Oct 12;20(10):e0014776. doi: 10.1371/journal.pntd.0014776 (PMC13712677; doi:10.1371/journal.pntd.0014776)
Supplement: Supplementary file 5 [file pntd.0014776.s008.docx]

### **Review Report**

**General assessment**

The study aims to identify, in a vulnerable community, the social and environmental factors associated with the distribution of *Aedes aegypti* and chikungunya. To achieve this, the authors employ a methodological approach integrating primary data from serological investigation, vector monitoring, and information collected through questionnaires and field observations, in addition to environmental data, all at a fine spatial scale. The study also applies robust statistical modelling, which gives the work relevant scientific value.

The authors identify socio-environmental factors related to the distribution of the disease and the vector in the studied community. However, the paper has some limitations that should be considered. Some important information is missing, and this makes it difficult to fully understand the study. Also, the results need to be discussed in more detail. Below, I give my comments by section

### **Title**

Although the title mentions “communities,” the study actually covers only one community, which limits the generalizability of the results (a limitation acknowledged by the authors themselves). Therefore, I suggest changing it to the singular form "community"

**Introduction**

- Add reference in: *“The proliferation of these mosquitoes is driven by environmental and socioeconomic factors that are typically present in lowincome and informal communities”.*
- The relationship between precipitation, mosquito presence, and the seasonal distribution of rainfall appears to be highly localized: “*The increase in mosquito breeding sites, both natural and artificial, results from water accumulation and improper water storage in households and the rise in rainfall during the first half of the year [9]*”

As it stands, the initial reading gives the impression that the pattern described is generalizable, which may not be the case.

### **Methods**

The study lacks a proper characterisation of the study area regarding population size and climatic conditions, both of which are relevant to the scientific question and to understanding mosquito-borne diseases.

There is insufficient information about which variables were included in the analysis. For instance, in the methods section, the authors mention that the questionnaire included questions on *employment status (formal or informal), household maximum income, number of residents, number of rooms, occurrence of water shortages, history of flooding, and garbage collection frequency*. However, none of these variables appear in the results, even in the descriptive section, despite their potential importance for the dynamics of disease and vector distribution.

In addition, in the table *“Individual and Household Characteristics of participants included in the study”*, there is a variable (*marital status*) that was not mentioned in the questionnaire description. The authors are encouraged to include the questionnaire in the supplementary material and to provide a justification for their choice of variables.

In the subsection describing the vector model, it is stated that “all relevant covariates were incorporated,” yet no details are provided on which covariates these were or how they were treated.

It is also unclear whether continuous variables were standardised, and the model specifications (including equations) are not presented.

The paper does not describe how participants were selected for the serological survey. Was recruitment done through a local health facility? Were households spatially stratified?

There is no exploratory analysis of the vector data, which would be essential, especially given that vector monitoring occurred for only 5–7 days in a single month, a period that may correspond to low mosquito density in several municipalities (though possibly not in Salvador). The vector dynamics in Salvador could be briefly addressed in the introduction. Reference 27, cited by the authors, may serve as a starting point.

The rationale for defining “observation of mosquitoes in the last seven days” should also be explained.

It is not entirely clear why Landsat imagery from 2016, 2018, and 2019 was used, as these years do not appear to correspond with the main study period.

### **Results**

In the *INLA-SPDE model for Ae. aegypti egg count in ovitraps*, there is a result for the variable *food insecurity*, which I could not trace back to its origin. It appears to be statistically significant, yet it is not highlighted in bold as the others, and it is not discussed in the text.

The manuscript mentions a correlation with *percentage of impervious surface land coverage* in the mosquito models and presents an IRR = 0.83 (95% CI: 0.66–1.03). This should be checked carefully.

The authors claim that including LST and mosquito data improves the performance of the *Chikungunya prevalence* model; however, no metrics are provided for the model without these variables, making it impossible to assess improvement.

In Figure 2 (map of seroprevalence), the source of the values is unclear. Based on the methods, I understood that the response variable was dichotomous (positive/negative). This reinforces the need to present the full model specification to avoid confusion.

### **Discussion**

Overall, the discussion of the results could be expanded, as it currently lacks theoretical depth and critical engagement with both national and international literature. Some examples include:

- Although the authors criticise traditional vector control methods, the practical measures they propose based on their findings remain within the same traditional framework (e.g., yard cleanup and elimination of breeding sites).
- The authors state that “fine-scale implementation of this method could help develop an entomological metric for identifying areas at higher transmission risk.” However, the study did not find a statistically significant relationship with egg counts, so the empirical basis for this statement is unclear.
- The discussion does not consider the possibility that infections may have occurred in other parts of the municipality. Urban mobility is an important factor not discussed by the authors and should be considered.
- Reference 27, cited in relation to vector abundance and temperature, does not provide evidence supporting that claim; this needs to be verified.
- The reported seroprevalence (42%) is not compared with previous studies from Salvador, Bahia, or other Brazilian regions.
- The authors argue that, under climate change, the impact in vulnerable areas could be even worse, considering that temperatures in these regions are already higher. However, it would be important to include a discussion noting that very high temperatures are not favourable to the vector; in fact, projections suggest that the impact of global warming on vector infestation may be greater in cooler regions of Brazil, such as the South ([Heath; Muniz Alves; Bonsall (2025)](https://www.zotero.org/google-docs/?broken=gnbCL4)
- The authors mention “adaptation measures aimed at reducing these temperature disparities.” What specific measures do they suggest? It would be useful to elaborate on this point and cite studies with relevant findings.
- The statement that “an important insight from this study is the relevance of high-resolution spatial estimation of *Ae. aegypti* eggs as a predictor of CHIKV prevalence in humans” is not supported by the results, given that no significant association was observed between vector abundance and human exposure.
- Remove repeated words in: underscore their value as **monitoring tool** monitoring tool [29,30] for health.

### **Writing and translation issues**

The manuscript needs grammatical and textual revision. There are many disconnected sentences, especially in the discussion section. In the Portuguese abstract (possibly automatically translated), numerous errors compromise comprehension and distort the content when compared to the English version.
