# Supplementary material for: Socio-environmental assessment of Aedes aegypti distribution and risk of chikungunya in vulnerable community
Source: PLoS Negl Trop Dis. 2026 Oct 12;20(10):e0014776. doi: 10.1371/journal.pntd.0014776 (PMC13712677; doi:10.1371/journal.pntd.0014776)
Supplement: Supplementary file 6 [file pntd.0014776.s010.docx]

The authors have addressed all points raised during the review, which has greatly improved the clarity of the manuscript. Nevertheless, some minor errors, inconsistencies, and general aspects still require revision, particularly in the Discussion. Specific comments are provided below:

### **Abstract**

- Typographical error in “Amon 888…”.
- Remove “61% female” from the abstract, as it disrupts the flow and is not discussed elsewhere in the manuscript.

### **Line 133**

- The second sentence starting with “It is necessary” lacks clarity and should be revised.

### **Line 168**

- The table title should be completed. It presents not only characteristics but also results from statistical models.

### **Line 191**

- Start by explicitly stating that this is the model in which the outcome is chikungunya exposure.

### **Lines 200–202**

- The expression “such as” may not be appropriate unless other variables were retained for the same reason.
- Clarify what threshold was used to define an improvement in model performance. A WAIC difference <1 in most models does not seem sufficient to support this claim.

### **Line 269-272**

- The sentence should be revised for clarity and flow.

### **Line 278**

- Replace strong claims with more cautious language (e.g., “may represent a risk to sustain transmission even when indoor control measures” instead of definitive statements).

### **Lines 281–282**

- Improve the connection between sentences for better coherence.

### **Line 284**

- A reference is required to support the statement regarding microclimatic conditions and preference for shaded/unpaved environments.

### **Lines 313–315**

- The term “productivity” is not appropriate here, as the outcome refers to abundance.
- The sentence requires revision for clarity and conceptual accuracy.

### **Lines 315–318**

- Verify the reference, as it does not appear to support the statement. [REF 28]

### **Statistical terminology**

- When reporting results from Bayesian models, I suggest replacing “significant” and “non-significant” with language more appropriate for Bayesian analysis, for example:
   *“The variable showed a positive association, with the 95% credible interval not including zero.”*

### **Line 367**

- *Aedes aegypti* should be italicised.

### **Lines 370–373**

- The statement should be better contextualised within the study.

### **Line 379**

- Clarify vulnerability (vulnerability to what?).

### **Lines 389- 393**

- The sentence is unclear and should be revised for clarity.

### **Line 397**

“..have been documented in other tropical and subtropical cities…”

- Add a reference to support the statement.

### **Lines 400–402**

- The statement is not well supported. It appears that the intended meaning is that increasing temperature, in locations where the mean temperature is already high, may not lead to increased vector abundance, potentially resulting in a plateau or even a decline. Please revise accordingly.

### **Line 410**

- The reference is relevant but not appropriately cited. It refers to *Anopheles* (with mention of *Culex*) and malaria. Consider generalising to “mosquitoes” instead of “arboviral diseases”.

### **Lines 576–578**

“This framework culminated in the estimation of a geostatistical spatial model using the Integrated 577 Nested Laplace Approximation (INLA)”

**Suggested revision:**
 *We fitted a geostatistical model using the Integrated Nested Laplace Approximation (INLA).*

**Supplementary material**

- Model 22 in Table S2 contains an extraneous “+” sign at the end.

## **General comments**

### **1**

There is some concern regarding the retention of land surface temperature and egg count variables in the models. Although the authors justified their inclusion based on WAIC (with extremely small differences, often <1), as well as biological plausibility and parsimony, it is possible that these variables were preferentially retained due to their well-established role in the vector-borne disease literature. However, at finer spatial scales, such as that considered in this study, there may be insufficient variability in these variables, which is reflected in greater uncertainty in the estimates. Therefore, caution is warranted when interpreting and discussing these effects.

### **2**

The discussion on how climate change may increase temperatures beyond optimal thresholds for the vector is somewhat unclear. This issue appears in multiple parts of the Discussion, sometimes as disconnected statements, sometimes without clear linkage to the study results, and occasionally in a contradictory manner. A careful revision of this section is recommended to improve coherence and avoid repetition.
