# Supplementary material for: Socio-environmental assessment of Aedes aegypti distribution and risk of chikungunya in vulnerable community
Source: PLoS Negl Trop Dis. 2026 Oct 12;20(10):e0014776. doi: 10.1371/journal.pntd.0014776 (PMC13712677; doi:10.1371/journal.pntd.0014776)
Supplement: Supplementary file 7 [file pntd.0014776.s011.docx]

**Reviewer's Responses to Questions**

### **Title**

Although the title mentions “communities,” the study actually covers only one community, which limits the generalizability of the results (a limitation acknowledged by the authors themselves). Therefore, I suggest changing it to the singular form "community"

We appreciate the comments of the reviewer. The title has been revised to use the singular form “community,” which more accurately reflects the scope of the study.

**Introduction**

Add reference in: *“The proliferation of these mosquitoes is driven by environmental and socioeconomic factors that are typically present in low-income and informal communities”.*

Done as suggested (line 112).

The relationship between precipitation, mosquito presence, and the seasonal distribution of rainfall appears to be highly localized: “*The increase in mosquito breeding sites, both natural and artificial, results from water accumulation and improper water storage in households and the rise in rainfall during the first half of the year [9]*”

As it stands, the initial reading gives the impression that the pattern described is generalizable, which may not be the case.

The manuscript has been updated following the reviewer’s suggested wording to better reflect the localized nature of the relationship between precipitation, mosquito presence, and seasonal rainfall patterns (lines 109-119).

### **Methods**

The study lacks a proper characterisation of the study area regarding population size and climatic conditions, both of which are relevant to the scientific question and to understanding mosquito-borne diseases.

The text was revised to improve the characterization of the study area, population size and climatic conditions (lines 460-465).

There is insufficient information about which variables were included in the analysis. For instance, in the methods section, the authors mention that the questionnaire included questions on *employment status (formal or informal), household maximum income, number of residents, number of rooms, occurrence of water shortages, history of flooding, and garbage collection frequency*. However, none of these variables appear in the results, even in the descriptive section, despite their potential importance for the dynamics of disease and vector distribution.

A detailed list of the variables included in the analyses was incorporated as supporting information (Sup 1) as well as a descriptive table showing univariable analyses.

In addition, in the table *“Individual and Household Characteristics of participants included in the study”*, there is a variable (*marital status*) that was not mentioned in the questionnaire description. The authors are encouraged to include the questionnaire in the supporting information and to provide a justification for their choice of variables.

Done as suggested.

In the subsection describing the vector model, it is stated that “all relevant covariates were incorporated,” yet no details are provided on which covariates these were or how they were treated.

The data analysis section was revised and restructured to improve clarity and consistency.

It is also unclear whether continuous variables were standardised, and the model specifications (including equations) are not presented.

All continuous covariates were standardized prior to model fitting using the scale() function in R, which centers each variable at its mean and rescales it by its standard deviation. This transformation results in covariates with mean zero and unit variance, and the standardized values were used in all model specifications. This explanation was included in the revised version (lines 587-589) as well as the model specifications.

The paper does not describe how participants were selected for the serological survey. Was recruitment done through a local health facility? Were households spatially stratified?

Participants were recruited through household visits conducted across the study area using a census-based sampling approach, rather than through a local health facility. All eligible residents within the well-defined study area who met the inclusion criteria were invited to participate. Therefore, we have now clarified in the Methods section that all residents within the study area who provided informed consent or assent had peripheral blood collected as part of the serological survey (lines 474-480)

There is no exploratory analysis of the vector data, which would be essential, especially given that vector monitoring occurred for only 5–7 days in a single month, a period that may correspond to low mosquito density in several municipalities (though possibly not in Salvador). The vector dynamics in Salvador could be briefly addressed in the introduction. Reference 27, cited by the authors, may serve as a starting point.

We have now included a descriptive and exploratory analysis of the vector data to address this concern. In Salvador, *Ae. aegypti* population dynamics differ from those observed in municipalities with stronger temperature seasonality. Due to relatively stable temperatures throughout the year, vector abundance and arbovirus transmission in Salvador are primarily driven by rainfall. Transmission intensity typically increases during the rainy season, which extends from April to August, with a marked peak between April and June. Therefore, vector monitoring conducted in May coincides with a period of high vector abundance and elevated arbovirus transmission risk in the city, as previously documented.

The rationale for defining “observation of mosquitoes in the last seven days” should also be explained.

The rationale for using “observation of mosquitoes in the last seven days” is to capture recent mosquito presence while minimizing recall bias. This time window is commonly used in household surveys and reflects short-term environmental and climatic conditions that influence mosquito activity. We have clarified this rationale in the Methods section (lines 493-495).

It is not entirely clear why Landsat imagery from 2016, 2018, and 2019 was used, as these years do not appear to correspond with the main study period.

Our objective was to derive a land surface temperature (LST) layer that adequately captured the spatial heterogeneity of thermal patterns within the study area, rather than to assess temporal variation. For this purpose, we used multiple Landsat images with less than 10% cloud cover to generate a representative LST surface. Because we were not interested in interannual or seasonal temperature dynamics, and because mean annual air temperature in Salvador shows limited variability across years, we considered it methodologically appropriate to include images acquired outside the main study period. Moreover, the availability of cloud-free Landsat scenes over the study area was limited, and extending the temporal window back to 2016 was necessary to obtain a sufficient number of images meeting the cloud-coverage criteria. This rationale has now been clarified in the Methods section (lines 513-516).

### **Results**

In the *INLA-SPDE model for Ae. aegypti egg count in ovitraps*, there is a result for the variable *food insecurity*, which I could not trace back to its origin. It appears to be statistically significant, yet it is not highlighted in bold as the others, and it is not discussed in the text.

We apologize for this unintended omission. We have now provided a clearer description of this explanatory covariate of its origin and measurement in the Supporting information Table 1 corrected its formatting in the results table and expanded the Results and discussion sections to explicitly report and interpret its statistically significant association.

The manuscript mentions a correlation with *percentage of impervious surface land coverage* in the mosquito models and presents an IRR = 0.83 (95% CI: 0.66–1.03). This should be checked carefully.

Following a detailed revision in response to the reviewers’ comments, we modified the model selection strategy to improve consistency and statistical appropriateness. A manual backward selection procedure was adopted since the beginning of the analysis, under which this variable was not retained in the final model.

The authors claim that including LST and mosquito data improves the performance of the *Chikungunya prevalence* model; however, no metrics are provided for the model without these variables, making it impossible to assess improvement.

We revised the model selection strategy and included a detailed table presenting the ranking of the candidate model sets (Supporting information Tables 1 and 2).

In Figure 2 (map of seroprevalence), the source of the values is unclear. Based on the methods, I understood that the response variable was dichotomous (positive/negative). This reinforces the need to present the full model specification to avoid confusion.

Although the response variable was dichotomous (positive/negative), we fitted a Bayesian geostatistical model with a binomial likelihood and a logit link using the INLA-SPDE framework, which enabled the estimation of a continuous spatial surface representing the underlying seroprevalence across the study area. This information was expanded in the method section.

### **Discussion**

Overall, the discussion of the results could be expanded, as it currently lacks theoretical depth and critical engagement with both national and international literature. Some examples include:

- Although the authors criticise traditional vector control methods, the practical measures they propose based on their findings remain within the same traditional framework (e.g., yard cleanup and elimination of breeding sites).

We would like to clarify that, while some of the actions discussed (e.g., yard cleanup and elimination of breeding sites) may resemble traditional vector control measures at the household level, our findings emphasize that the effectiveness and sustainability of these actions are fundamentally constrained by broader structural conditions. In particular, inadequate provision of basic urban services, such as regular solid waste collection, reliable water supply, and adequate drainage infrastructure, limits residents’ ability to maintain clean peridomestic environments and reduce waste accumulation.

Therefore, rather than reinforcing a purely individual- or household-centered approach, our study support the need for integrated interventions that combine community engagement with improvements in essential urban services. Strengthening waste management systems and water and sanitation services is critical to enabling and sustaining household-level practices, and to addressing the structural drivers of vector proliferation. We have revised the discussion to better highlight this distinction and to underscore that effective vector control in vulnerable urban settings requires coordinated action beyond traditional, behavior-focused measures.

- The authors state that “fine-scale implementation of this method could help develop an entomological metric for identifying areas at higher transmission risk.” However, the study did not find a statistically significant relationship with egg counts, so the empirical basis for this statement is unclear.

We agree that the association between egg counts and the CHIKV exposure outcome did not reach conventional levels of statistical significance in this study. However, the variable remained relevant within the multivariable modeling framework, which suggests that it may still capture meaningful underlying ecological variation.

Due to our analysis focused on prevalence rather than incidence, which may attenuate associations with contemporaneous entomological indicators such as egg counts. In addition, egg abundance is influenced by external factors, including vector control activities, temporal mismatches between entomological sampling and infection events, and environmental disturbances, that may obscure direct statistical associations in cross-sectional analyses.

On this basis, we view our findings as hypothesis-generating rather than definitive. We have clarified in the revised manuscript that further studies specifically designed to analyze incidence data, with tighter temporal alignment between entomological and epidemiological measurements and reduced external interference, are needed to better assess the utility of fine-scale egg count–based metrics for identifying areas of higher transmission risk.

- The discussion does not consider the possibility that infections may have occurred in other parts of the municipality. Urban mobility is an important factor not discussed by the authors and should be considered.

We have revised the discussion to explicitly acknowledge that infections may have occurred outside the area of residence (lines 435-437)

- Reference 27, cited in relation to vector abundance and temperature, does not provide evidence supporting that claim; this needs to be verified.

We have carefully revised the text and verified the cited reference. We acknowledge that Reference 27 (now, reference 20) does not provide evidence on vector abundance and temperature; rather, it reports an association between chikungunya incidence and temperature. The text has been corrected accordingly (line 380-382).

- The reported seroprevalence (42%) is not compared with previous studies from Salvador, Bahia, or other Brazilian regions.

We have added a paragraph at the beginning of the Discussion comparing the 42% seroprevalence observed in our study with previous reports from Salvador and other Brazilian regions, to better contextualize the magnitude of exposure observed in this vulnerable community (lines 257-265).

- The authors argue that, under climate change, the impact in vulnerable areas could be even worse, considering that temperatures in these regions are already higher. However, it would be important to include a discussion noting that very high temperatures are not favourable to the vector; in fact, projections suggest that the impact of global warming on vector infestation may be greater in cooler regions of Brazil, such as the South ([Heath; Muniz Alves; Bonsall (2025)](https://www.zotero.org/google-docs/?broken=gnbCL4)

We have expanded the Discussion as suggested (lines 386-402)

- The authors mention “adaptation measures aimed at reducing these temperature disparities.” What specific measures do they suggest? It would be useful to elaborate on this point and cite studies with relevant findings.

We have expanded the discussion to address this point in greater detail. Specifically, we now outline adaptation measures aimed at reducing temperature disparities, including the implementation of green corridors, the expansion of vegetated areas, and the use of thermal mitigation strategies such as reflective (thermal) paints and insulated or plastic wall materials. In addition, we have incorporated citations into relevant studies that report empirical evidence on the effectiveness of these measures in reducing ambient and surface temperatures (lines 405-413).

- The statement that “an important insight from this study is the relevance of high-resolution spatial estimation of *Ae. aegypti* eggs as a predictor of CHIKV prevalence in humans” is not supported by the results, given that no significant association was observed between vector abundance and human exposure.

We agree that, in this study, the association between *Ae. aegypti* egg counts and CHIKV exposure did not reach conventional levels of statistical significance. We have therefore revised the text to avoid any overstatement of this relationship. However, we note that substantial spatial heterogeneity in seroprevalence was observed, and that egg abundance remained informative within the multivariable modeling framework, suggesting it may still capture relevant underlying ecological variations not fully explained by other covariates. On this basis, we have reframed our interpretation to emphasize the potential value of high-resolution spatial entomological data for future research. Specifically, we highlight the need for studies conducted at different spatial resolutions to support the development and validation of entomological indices capable of predicting arbovirus transmission patterns at the community scale (lines 373-379)

- Remove repeated words in: underscore their value as **monitoring tool** monitoring tool [29,30] for health.

*Done as suggested.*

### **Writing and translation issues**

The manuscript needs grammatical and textual revision. There are many disconnected sentences, especially in the discussion section. In the Portuguese abstract (possibly automatically translated), numerous errors compromise comprehension and distort the content when compared to the English version.

*Done as suggested.*

***Methods***

Reviewer #1: The objectives are clear and the study design is appropriate. I am not qualified to comment on the modelling. However, I have concerns about the statistical analysis, and specifically whether there any adjustment for multiple tests when looking for correlations between variables and exposure (see Results section of review).

Some more references should be added where suitable. Was the September 2022 survey done for this study or was it conducted by others and if so is there a reference for it? Is there a reference for the Epanechnikov kernel function?

The survey conducted in September 2022 was carried out by the field team as part of the activities of the present study (lines 487-488). The text has been revised to avoid misunderstandings. In addition, a reference for the Epanechnikov kernel function has been added, as suggested (lines 536).

Provide details of the ELISA kit – was it made by EUROIMMUN and where are they based?

We added details about the ELISA kit in lines 545-546.

Reviewer #2: The objectives of the study are clearly articulated, and a testable hypothesis is stated. The study design is appropriate to address these objectives, and the target population is well described and suitable for the hypothesis being tested. The statistical analyses used are appropriate. Ethical and regulatory requirements appear to have been met, as the manuscript includes a statement of ethics committee approval.
However, the manuscript does not provide sufficient exploratory details to assess whether the mosquito sample size is adequate to support the conclusions. I recommend that the authors further analyze these data and include the corresponding results in the manuscript. There is also a lack of clarity regarding the origin and selection of the variables and data presented. Providing more detailed information on how these were chosen and obtained would improve the transparency and reproducibility of the study. Recommend: major revision

*Done as suggested.*

**Results**

Reviewer #1: The authors report in the Results section that they found a significant association between advanced age and exposure. This had a p-value of 0.022. However, with so many comparisons one would expect some to be ‘significant’ (with a p-value of 0.05 or less) just by chance. To account for all these tests the authors should apply some type of correction such as Bonferroni’s adjustment.

The p-values reported in the Results section correspond exclusively to exploratory univariable analyses and were not intended for confirmatory inference. These analyses were used as an initial descriptive screening step. The study’s conclusions and discussion are based on the final spatial INLA-SPDE model, in which all covariates were evaluated jointly within a Bayesian framework. The exploratory univariable results are presented for completeness and transparency, and their relevance was subsequently evaluated and supported in the final spatial model. In this context, we consider that additional multiple-comparison corrections, such as Bonferroni adjustment, are not required.

Reviewer #2: While the manuscript presents relevant results, there are several issues that impact the clarity and completeness of the analysis. In the INLA-SPDE model for Aedes aegypti egg counts, the variable representing food insecurity appears to be statistically significant, but its origin is unclear, it is not highlighted as other significant variables are, and it is not discussed in the text. Additionally, the reported association with impervious surface coverage (IRR = 0.83; 95% CI: 0.66–1.03) should be carefully reviewed.
The authors state that including LST and mosquito data improves the Chikungunya model's performance, but they do not provide comparative metrics to support this claim. This limits the reader’s ability to evaluate whether the inclusion of these variables truly improves model fit.
Furthermore, in Figure 2, which shows a map of seroprevalence, the source of the values is unclear. The methods suggest that the outcome variable is binary (positive/negative), which makes it difficult to interpret the mapped values. This highlights the importance of providing a full model specification and clear explanation of derived outputs to avoid confusion. Recommend: major revision

We appreciate the reviewer’s insightful comments. These points have been addressed above, in response to the Editor’s comments.

**Conclusions**

Reviewer #1: The Discussion is quite basic and has limited reference to other studies. For example, it concludes that control actions should be focused on ‘the peridomestic environment, such as cleanup campaigns and the elimination of breeding sites, which may be effective in reducing vector density in affected areas. The importance of peridomestic activities is thoroughly well known in the field (see the CDC’s Surveillance and Control of Aedes aegypti and Aedes albopictus in the United States) and is not a novel finding.

We agree that the importance of peridomestic environments for *Aedes* control is well established and not novel. We have revised the Discussion to better reference existing literature and guidelines and to clarify that the contribution of our study lies in highlighting fine-scale spatial heterogeneity to help prioritize where these well-known control actions may be most effectively targeted (lines 295-306)

There is reference to ‘a study conducted in a vulnerable community in Salvador, Bahia, Brazil, which reported that structural deficiencies in both housing and surrounding streets were associated with prior CHIKV infection’ by Anjos et al.. Given that the submitted article and the paper by Anjos were both considering chikungunya exposure in the same city, are there other similarities or differences that can be discussed here?

We thank the reviewer for this pertinent suggestion. We have expanded the Discussion to more explicitly compare our findings with those of Anjos et al., highlighting both similarities and differences (lines 343-346). While both studies identify structural and environmental vulnerabilities associated with chikungunya exposure in Salvador, Anjos et al. examined patterns at broader neighborhood scales using socio-structural indicators, whereas our study integrates serological, entomological, and environmental data at a finer spatial resolution within a single informal settlement. This comparison has been added to clarify how our results complement and extend previous findings in the same urban context.

There is discussion of a trend between the risk of exposure to chikungunya and the predicted Ae. aegypti egg count. The map shows a large exposure hotspot that did not have particularly high egg counts. Is there anything to discuss here? Perhaps this could be pointed out as an example of where
egg counts do not accurately reflect the true abundance of adults (the vectorial stage).

We thank the reviewer for this observation. We have added a brief discussion noting the spatial mismatch between chikungunya exposure hotspots and predicted egg counts and highlighting that ovitraps measure oviposition activity rather than adult mosquito abundance, which may explain why egg counts do not always reflect transmission risk (lines 389-394).

The temperature profile of the vulnerable communities is interesting, and the authors make an important point about potential increases in risk with climate change. Are there other studies supporting this finding at a sub-city level?

We thank the reviewer for this comment. We have added references in the Discussion to studies showing that intra-urban thermal heterogeneity is associated with *Aedes* ecology and arboviral risk at sub-city scales (lines 428-432), strengthening the interpretation of our findings in the context of climate change.

Overall I think the authors need to do more to show how their findings are useful.

We thank the reviewer for this comment. We clarify the practical relevance of our findings in the Discussion and concluding paragraph by explicitly linking fine-scale risk mapping to targeted, micro-hotspot–oriented vector control and community-based interventions (a precision public health approach), including prioritization of peridomestic areas, waste management, and neighborhood-specific actions informed by integrated entomological, environmental, and social data.

Reviewer #2: Although some of the conclusions are supported by the analyses and results presented, one point that stands out is the authors’ claim that fine-scale spatial data on the vector could serve as an indicator for chikungunya transmission. This statement is not supported by the findings of the study. Additionally, the manuscript lacks a discussion on the limitations of the vector sampling, which was conducted over a short period (one week). This is particularly relevant given that Aedes aegypti populations are highly influenced by climatic factors, and the short sampling window may have significantly affected the observed results. Although the authors criticise traditional vector control methods, the practical measures they propose based on their findings remain within the same traditional framework (e.g., yard cleanup and elimination of breeding sites)

We thank the reviewer for these important comments. We have revised the manuscript to clarify that fine-scale entomological data should not be interpreted as a direct proxy for chikungunya transmission, but rather as one component within an integrated risk framework. We also expanded the Discussion to explicitly acknowledge the limitations of the short ovitraps sampling period and the influence of climatic variability on *Ae. aegypti* populations. Finally, we clarified that our recommendations do not propose new control tools per se, but emphasize the targeted, fine-scale prioritization of traditional vector control actions based on spatial risk patterns.

**Editorial and Data Presentation Modifications?**


Reviewer #1: Minor comments
Mosquitoes should not be italicized.
Chikungunya should not be spelled with a capital letter except at the start of a sentence.
*Done as suggested.*

Line 1: CHIKV refers to the virus, not the disease. It might be clearer to write ‘Chikungunya fever, caused by chikungunya virus (CHIKV), has emerged…’
*Done as suggested.*

‘Hotspots for Ae. aegypti breeding.’ Strictly the authors are looking for larval development sites not places where the mosquitoes breed (mate).
*Done as suggested.*

In Table 1, a variable is labelled as ‘Race,’ but the Methods describe this as ‘self-reported skin color.’ For consistency and clarity, I recommend the authors use same term (‘self-reported skin color’) in the table.

*Done as suggested.*

**Summary and General Comments**

Reviewer #1: This study uses a combination of serological assays, mosquito trapping, socioeconomic surveys and land use data to investigate high risk areas of chikungunya exposure in vulnerable communities of Salvador, Brazil. The article is well written, and seeks to identify areas that might require additional vector control activities, particularly in the context of climate change which could lead to increased vector populations. Although the study could prove valuable in this context, I felt that the manuscript led readers to think that there is a lack of studies of this type, and aside from the useful maps it came to some rather expected conclusions.

We agree that there is an extensive body of literature examining socio-environmental drivers of Aedes-borne arboviruses, and we did not intend to suggest that studies of this type are lacking. We have revised the Introduction to better acknowledge the breadth of existing work and to more clearly position our contribution in relation to this literature. Specifically, we clarify that the novelty of our study lies not in identifying socio-environmental risk factors per se, but in integrating serological, entomological, and environmental data at a fine spatial scale within a single vulnerable community, allowing the identification of micro-scale heterogeneity and localized hotspots that are often obscured in broader-scale analyses. We also revised the Discussion to more explicitly frame our findings as expected but epidemiologically important, and to emphasize their value for guiding precision, context-specific vector control and adaptation strategies in the context of climate change.

In the Introduction, why not reference the review by Whiteman et al 2020 (Do socioeconomic factors drive Aedes mosquito vectors and their arboviral diseases: A systematic review of dengue, chikungunya, yellow fever, and Zika virus)? That authors of that review included almost one hundred studies in their analysis, showing the abundance of previous work on this subject.

*Done as suggested (lines 112-114)*

Reviewer #2: The study aims to identify, in a vulnerable community, the social and environmental factors associated with the distribution of Aedes aegypti and chikungunya. To achieve this, the authors employ a methodological approach integrating primary data from serological investigation, vector monitoring, and information collected through questionnaires and field observations, in addition to environmental data, all at a fine spatial scale. The study also applies robust statistical modelling, which gives the work relevant scientific value.
The authors identify socio-environmental factors related to the distribution of the disease and the vector in the studied community. However, the paper has some limitations that should be considered. Some important information is missing, and this makes it difficult to fully understand the study. Also, the results need to be discussed in more detail.

We agree that additional information and a more detailed discussion improve the clarity and interpretation of the manuscript. In response, we revised we clarified missing methodological details in the Methods (Data collection and Data analysis), expanded the description of the statistical models, and added a dedicated limitations paragraph in the Discussion. We also expanded the Discussion to provide greater context and interpretation of the results and their implications for targeted interventions.
