# Supplementary material for: Socio-environmental assessment of Aedes aegypti distribution and risk of chikungunya in vulnerable community
Source: PLoS Negl Trop Dis. 2026 Oct 12;20(10):e0014776. doi: 10.1371/journal.pntd.0014776 (PMC13712677; doi:10.1371/journal.pntd.0014776)
Supplement: Supplementary file 8 [file pntd.0014776.s012.docx]

**June 9, 2026**

**Editorial Board**

*PLOS Neglected Tropical Diseases*

**Re: Response to Reviewers**

**Manuscript Title:** *Socio-environmental assessment of Aedes aegypti distribution and risk of chikungunya in a vulnerable community*

Dear Editors and Reviewers,

We appreciate the reviewers’ thoughtful and constructive feedback, and we are grateful for their recognition of the value of our work.

As requested, we have responded to each reviewer comment point by point. To facilitate review, our responses were provided in blue italic font, clearly distinguishing them from the original reviewer comments.

Sincerely,

Hussein Khalil, PhD, on behalf of the authors

**Journal Requirements:**

We have noticed that you have uploaded Supporting Information files, but you have not included a complete list of legends. Please add a full list of legends for your Supporting Information files ("Database_CHIKV_MR.csv and "Database_ovitraps.xlsx") after the references list.

*Done as suggested, a full list of legends was added. (Tables_Legend)*

**Reviewers' Comments:**

**Reviewer #1:** The authors have carefully addressed the comments from the original review.

A few minor points: In the their response to reviewer comments, the authors state that "We have clarified in the revised manuscript that further studies specifically designed to analyze incidence data, with tighter temporal alignment between entomological and epidemiological measurements and reduced external interference, are needed to better assess the utility of fine scale egg count–based metrics for identifying areas of higher transmission risk. However, I cannot find this reference to incidence data.

*We apologize for the lack of clarity. While we intended to address this point in the Discussion, the revised text did not explicitly refer to incidence data as indicated in our response. To correct this, we have now added the statement to the Discussion section. (Lines 425-430).*

References are provided later in the Discussion but I feel Line 370 requires a reference.

*Done as suggested.*

The authors should carefull check for consistent use of italics (Line 367) and abbreviation of Aedes genus name (Line 391).

*Done as suggested.*

## **Reviewer #2:**

The authors have addressed all points raised during the review, which has greatly improved the clarity of the manuscript. Nevertheless, some minor errors, inconsistencies, and general aspects still require revision, particularly in the Discussion. Specific comments are provided below:

*We sincerely thank you for your thoughtful comments and constructive suggestions, which have significantly contributed to improving the quality and clarity of our manuscript. We have carefully considered each recommendation and incorporated the corresponding revisions throughout the manuscript.*

### **Abstract**

- Typographical error in “Amon 888…”.
- Remove “61% female” from the abstract, as it disrupts the flow and is not discussed elsewhere in the manuscript~~.~~

*Dones as suggested.*

### **Line 133**

- The second sentence starting with “It is necessary” lacks clarity and should be revised.

*The sentence has been revised to improve clarity. It now reads: “Integrated policies are needed to promote social mobilization, health and environmental education, access to adequate housing and sanitation, and prevent deforestation” (Lines 124-126).*

### **Line 168**

- The table title should be completed. It presents not only characteristics but also results from statistical models.

*We have revised the table title to better reflect its content, which includes not only descriptive characteristics but also results from statistical models. (Lines 143-144)*

### **Line 191**

- Start by explicitly stating that this is the model in which the outcome is chikungunya exposure.

*We have revised the sentence to explicitly state that chikungunya exposure is the outcome of the model. (Lines 167-168)*

### **Lines 200–202**

- The expression “such as” may not be appropriate unless other variables were retained for the same reason.

*We have revised the sentence to remove the expression “such as” and clarify the role of the variables in the model. (Lines 178-179).*

- Clarify what threshold was used to define an improvement in model performance. A WAIC difference <1 in most models does not seem sufficient to support this claim.

*Models with ΔWAIC < 2 were considered to have comparable predictive performance; however, final model selection was guided by both statistical performance and biological interpretability. When competing models exhibited similar WAIC values, preference was given to the more parsimonious model, provided that it yielded ecologically meaningful parameter estimates and that the estimated effects of covariates were consistent with current biological understanding of the system.*

*We have revised the manuscript to clarify that the final model was selected based on both model performance and biological relevance, rather than on small differences in WAIC alone. (Lines 178-185).*

### **Line 269-272**

- The sentence should be revised for clarity and flow.

*We have revised the sentence to improve clarity and flow. The text was restructured to correct grammatical issues and to improve the coherence and logical progression of the ideas presented. (Lines 283-289).*

### **Line 278**

- Replace strong claims with more cautious language (e.g., “may represent a risk to sustain transmission even when indoor control measures” instead of definitive statements).

*Done as suggested (Lines 252-258).*

### **Lines 281–282**

- Improve the connection between sentences for better coherence.

*Done as suggested. (Lines 265-266).*

### **Line 284**

- A reference is required to support the statement regarding microclimatic conditions and preference for shaded/unpaved environments.

*Done as suggested. (Line 269).*

### **Lines 313–315**

- The term “productivity” is not appropriate here, as the outcome refers to abundance.
- The sentence requires revision for clarity and conceptual accuracy.

*Done as suggested. (Lines 297-300).*

### **Lines 315–318**

- Verify the reference, as it does not appear to support the statement. [REF 28]

*We have added a new reference to support the statement regarding microclimatic conditions and the preference for shaded and unpaved environments. (Line 303).*

### **Statistical terminology**

- When reporting results from Bayesian models, I suggest replacing “significant” and “non-significant” with language more appropriate for Bayesian analysis, for example:
   *“The variable showed a positive association, with the 95% credible interval not including zero.”*

*The Results section was revised to ensure that the terminology and interpretation of findings are consistent with a Bayesian analytical approach. (Lines 153-164 and 170-178)*

### **Line 367**

- *Aedes aegypti* should be italicised.

*We have revised the manuscript to ensure that Aedes aegypti is consistently italicised throughout the text.*

### **Lines 370–373**

- The statement should be better contextualised within the study.

*We have revised the text to better contextualize the statement within the scope and findings of the present study. We also removed broader contextual information that was not directly supported by our results, ensuring that the discussion remains focused on the evidence generated by this study and its implications. (Lines 374-391)*

### **Line 379**

- Clarify vulnerability (vulnerability to what?).

*We have revised the sentence to clarify the outcome of interest, explicitly specifying vulnerability to chikungunya infection. (Lines 370-371)*

### **Lines 389- 393**

- The sentence is unclear and should be revised for clarity.

*Done as suggested. (Lines 377-382).*

### **Line 397**

“..have been documented in other tropical and subtropical cities…”

- Add a reference to support the statement.

*Done as suggested. (Line 384).*

### **Lines 400–402**

- The statement is not well supported. It appears that the intended meaning is that increasing temperature, in locations where the mean temperature is already high, may not lead to increased vector abundance, potentially resulting in a plateau or even a decline. Please revise accordingly.

### *The text was reworded to avoid potential confusion and to provide a more cautious interpretation of the relationship between temperature and vector abundance. (Lines 387-391).*

### **Line 410**

- The reference is relevant but not appropriately cited. It refers to *Anopheles* (with mention of *Culex*) and malaria. Consider generalising to “mosquitoes” instead of “arboviral diseases”.

*We have revised the text to better align with the scope of the cited reference, generalising the terminology from “arboviral diseases” to “mosquito-borne diseases (Lines 396).*

### **Lines 576–578**

“This framework culminated in the estimation of a geostatistical spatial model using the Integrated Nested Laplace Approximation (INLA)”

**Suggested revision:**

*We fitted a geostatistical model using the Integrated Nested Laplace Approximation (INLA).*

*Done as suggested (Lines 567-569)*

**Supplementary material**

### As the coordinates are included in the supplementary material (Database_CHIKV_MR), I am concerned about the potential identification of participants’ households.

*The household coordinates were included in the previous version of the supplementary dataset solely to facilitate the review process and allow reviewers to assess the spatial analyses. To further protect participant confidentiality, we have removed all geographic coordinates from the supplementary material in the revised version of the manuscript.*

- Model 22 in Table S2 contains an extraneous “+” sign at the end.

*The “+” was removed*

## **General comments**

### **1**

There is some concern regarding the retention of land surface temperature and egg count variables in the models. Although the authors justified their inclusion based on WAIC (with extremely small differences, often <1), as well as biological plausibility and parsimony, it is possible that these variables were preferentially retained due to their well-established role in the vector-borne disease literature. However, at finer spatial scales, such as that considered in this study, there may be insufficient variability in these variables, which is reflected in greater uncertainty in the estimates. Therefore, caution is warranted when interpreting and discussing these effects.

*We thank the reviewer for this thoughtful comment and agree that caution is warranted when interpreting the effects of land surface temperature and egg count in our models. We have revised the manuscript to avoid overstating their importance and to clearly acknowledge the uncertainty associated with these estimates.*

*Our decision to retain these variables was not based solely on their established role in the vector-borne disease literature. Rather, it was motivated by both their biological relevance and the substantial spatial heterogeneity observed within the study area. Despite the relatively fine spatial scale of the analysis, land surface temperature varied by approximately 5°C across the community (ranging from 28°C to 33°C), while predicted spatial egg counts varied more than threefold (approximately 25 to 127 eggs). Likewise, predicted seroprevalence showed marked spatial heterogeneity, ranging from approximately 15% to 74% across the study area. These patterns suggest that meaningful environmental and entomological gradients exist even within this relatively small urban setting.*

*At the same time, we recognize that the uncertainty surrounding the estimated effects limits the strength of inference that can be drawn regarding their independent contributions. Consequently, we have revised the text to emphasize that these variables should be interpreted as potentially relevant ecological correlates rather than definitive drivers of infection risk. Their retention in the final model reflects a balance between model performance, biological plausibility, and the observed fine-scale spatial variability, while acknowledging the uncertainty in their estimated effects. This information was included in the results section (Lines 178-185) and in the discussion (Lines 352-364)*

### **2**

The discussion on how climate change may increase temperatures beyond optimal thresholds for the vector is somewhat unclear. This issue appears in multiple parts of the Discussion, sometimes as disconnected statements, sometimes without clear linkage to the study results, and occasionally in a contradictory manner. A careful revision of this section is recommended to improve coherence and avoid repetition.

*We have substantially revised the Discussion to improve coherence, reduce redundancy, and avoid statements regarding climate change without direct linked to our results and their interpretation.*
