# Supplementary material for: Socio-environmental assessment of Aedes aegypti distribution and risk of chikungunya in vulnerable community
Source: PLoS Negl Trop Dis. 2026 Oct 12;20(10):e0014776. doi: 10.1371/journal.pntd.0014776 (PMC13712677; doi:10.1371/journal.pntd.0014776)
Supplement: Peer review history

# Peer review: Decision Letter 0

**DOI:** 10.1371/journal.pntd.0014776.r001

**Authors:** Paul Mireji, Clarence Mang'era

18 Dec 2025

Socio-environmental assessment of Aedes aegypti distribution and risk of Chikungunya in vulnerable communities.

PLOS Neglected Tropical Diseases

Dear Dr. Khalil,

Thank you for submitting your manuscript to PLOS Neglected Tropical Diseases. After careful consideration, we feel that it has merit but does not fully meet PLOS Neglected Tropical Diseases's publication criteria as it currently stands. Therefore, we invite you to submit a revised version of the manuscript that addresses the points raised during the review process.

Please submit your revised manuscript within by Feb 16 2026 11:59PM. If you will need more time than this to complete your revisions, please reply to this message or contact the journal office at plosntds@plos.org. When you're ready to submit your revision, log on to https://www.editorialmanager.com/pntd/ and select the 'Submissions Needing Revision' folder to locate your manuscript file.

Please include the following items when submitting your revised manuscript:

* A letter that responds to each point raised by the editor and reviewer(s). You should upload this letter as a separate file labeled 'Response to Reviewers'. This file does not need to include responses to any formatting updates and technical items listed in the 'Journal Requirements' section below.

* A marked-up copy of your manuscript that highlights changes made to the original version. You should upload this as a separate file labeled 'Revised Manuscript with Track Changes'.

* An unmarked version of your revised paper without tracked changes. You should upload this as a separate file labeled 'Manuscript'.

If you would like to make changes to your financial disclosure, competing interests statement, or data availability statement, please make these updates within the submission form at the time of resubmission. Guidelines for resubmitting your figure files are available below the reviewer comments at the end of this letter.

We look forward to receiving your revised manuscript.

Kind regards,

Clarence Mang'era, PhD

Academic Editor

PLOS Neglected Tropical Diseases

Paul Mireji

Section Editor

PLOS Neglected Tropical Diseases

Shaden Kamhawi

co-Editor-in-Chief

PLOS Neglected Tropical Diseases

orcid.org/0000-0003-4304-636XX

Paul Brindley

co-Editor-in-Chief

PLOS Neglected Tropical Diseases

orcid.org/0000-0003-1765-0002

**Additional Editor Comments:**

The manuscript requires revision to address several key areas that will enhance its readability and overall impact.

In addition to the reviewers' comments, please provide more details on the study's sample size. Also, the operational definitions of key variables need greater clarity. For example, concerning the measure "observation of mosquitoes in the last seven days," please clarify whether participants were asked to identify the specific vector species relevant to the study's objectives. Finally, the discussion section should be strengthened by ensuring it is thoroughly data-driven, connecting the findings to their public health relevance, and including an acknowledgement of the study's limitations. Remember to include line numbers in the revised MS.

**Journal Requirements:**

If the reviewer comments include a recommendation to cite specific previously published works, please review and evaluate these publications to determine whether they are relevant and should be cited. There is no requirement to cite these works unless the editor has indicated otherwise.

1) Please ensure that the CRediT author contributions listed for every co-author are completed accurately and in full.

At this stage, the following Authors/Authors require contributions: JAQUELINE CRUZ. Please ensure that the full contributions of each author are acknowledged in the "Add/Edit/Remove Authors" section of our submission form.

The list of CRediT author contributions may be found here: https://journals.plos.org/plosntds/s/authorship#loc-author-contributions

2) Please provide an Author Summary. This should appear in your manuscript between the Abstract (if applicable) and the Introduction, and should be 150-200 words long. The aim should be to make your findings accessible to a wide audience that includes both scientists and non-scientists. Sample summaries can be found on our website under Submission Guidelines:

https://journals.plos.org/plosntds/s/submission-guidelines#loc-parts-of-a-submission

3) Please upload all main figures as separate Figure files in .tif or .eps format. For more information about how to convert and format your figure files please see our guidelines:

https://journals.plos.org/plosntds/s/figures

4) Some material included in your submission may be copyrighted. According to PLOSu2019s copyright policy, authors who use figures or other material (e.g., graphics, clipart, maps) from another author or copyright holder must demonstrate or obtain permission to publish this material under the Creative Commons Attribution 4.0 International (CC BY 4.0) License used by PLOS journals. Please closely review the details of PLOSu2019s copyright requirements here: PLOS Licenses and Copyright. If you need to request permissions from a copyright holder, you may use PLOS's Copyright Content Permission form.

Please respond directly to this email and provide any known details concerning your material's license terms and permissions required for reuse, even if you have not yet obtained copyright permissions or are unsure of your material's copyright compatibility. Once you have responded and addressed all other outstanding technical requirements, you may resubmit your manuscript within Editorial Manager.

Potential Copyright Issues:

i) Figures 1, and 2. 3. Please (a) provide a direct link to the base layer of the map (i.e., the country or region border shape) and ensure this is also included in the figure legend; and (b) provide a link to the terms of use / license information for the base layer image or shapefile. We cannot publish proprietary or copyrighted maps (e.g. Google Maps, Mapquest) and the terms of use for your map base layer must be compatible with our CC BY 4.0 license.

Note: if you created the map in a software program like R or ArcGIS, please locate and indicate the source of the basemap shapefile onto which data has been plotted.

If your map was obtained from a copyrighted source please amend the figure so that the base map used is from an openly available source. Alternatively, please provide explicit written permission from the copyright holder granting you the right to publish the material under our CC BY 4.0 license.

If you are unsure whether you can use a map or not, please do reach out and we will be able to help you. The following websites are good examples of where you can source open access or public domain maps:

* U.S. Geological Survey (USGS) - All maps are in the public domain. (http://www.usgs.gov)

* PlaniGlobe - All maps are published under a Creative Commons license so please cite u201cPlaniGlobe, http://www.planiglobe.com, CC BY 2.0u201d in the image credit after the caption. (http://www.planiglobe.com/?lang=enl)

* Natural Earth - All maps are public domain. (http://www.naturalearthdata.com/about/terms-of-use/).

5) In the online submission form, you indicated that Researchers who wish to access the data can contact the data manager at the Oswaldo Cruz Foundation, Nivison Junior (nivisonjr@hotmail.com). All PLOS journals now require all data underlying the findings described in their manuscript to be freely available to other researchers, either

1. In a public repository

2. Within the manuscript itself

3. Uploaded as supplementary information.

This policy applies to all data except where public deposition would breach compliance with the protocol approved by your research ethics board. If your data cannot be made publicly available for ethical or legal reasons (e.g., public availability would compromise patient privacy), please explain your reasons by return email and your exemption request will be escalated to the editor for approval. Your exemption request will be handled independently and will not hold up the peer review process, but will need to be resolved should your manuscript be accepted for publication. One of the Editorial team will then be in touch if there are any issues.

6) Please amend your detailed Financial Disclosure statement. This is published with the article. It must therefore be completed in full sentences and contain the exact wording you wish to be published.

1) State the initials, alongside each funding source, of each author to receive each grant. For example: "This work was supported by the National Institutes of Health (####### to AM; ###### to CJ) and the National Science Foundation (###### to AM)."

2) State what role the funders took in the study. If the funders had no role in your study, please state: "The funders had no role in study design, data collection and analysis, decision to publish, or preparation of the manuscript."

3) If any authors received a salary from any of your funders, please state which authors and which funders..

If you did not receive any funding for this study, please simply state: u201cThe authors received no specific funding for this work.u201d

**Reviewers' Comments:**

Reviewer's Responses to Questions

**Key Review Criteria Required for Acceptance?**

As you describe the new analyses required for acceptance, please consider the following:

**Methods**

-Are the objectives of the study clearly articulated with a clear testable hypothesis stated?

-Is the study design appropriate to address the stated objectives?

-Is the population clearly described and appropriate for the hypothesis being tested?

-Is the sample size sufficient to ensure adequate power to address the hypothesis being tested?

-Were correct statistical analysis used to support conclusions?

-Are there concerns about ethical or regulatory requirements being met?

Reviewer #1: The objectives are clear and the study design is appropriate. I am not qualified to comment on the modelling. However, I have concerns about the statistical analysis, and specifically whether there any adjustment for multiple tests when looking for correlations between variables and exposure (see Results section of review).

Some more references should be added where suitable. Was the September 2022 survey done for this study or was it conducted by others and if so is there a reference for it? Is there a reference for the Epanechnikov kernel function?

Provide details of the ELISA kit – was it made by EUROIMMUN and where are they based?

Reviewer #2: The objectives of the study are clearly articulated, and a testable hypothesis is stated. The study design is appropriate to address these objectives, and the target population is well described and suitable for the hypothesis being tested. The statistical analyses used are appropriate. Ethical and regulatory requirements appear to have been met, as the manuscript includes a statement of ethics committee approval.

However, the manuscript does not provide sufficient exploratory details to assess whether the mosquito sample size is adequate to support the conclusions. I recommend that the authors further analyze these data and include the corresponding results in the manuscript. There is also a lack of clarity regarding the origin and selection of the variables and data presented. Providing more detailed information on how these were chosen and obtained would improve the transparency and reproducibility of the study. Recommend: major revision

**********

**Results**

-Does the analysis presented match the analysis plan?

-Are the results clearly and completely presented?

-Are the figures (Tables, Images) of sufficient quality for clarity?

Reviewer #1: The authors report in the Results section that they found a significant association between advanced age and exposure. This had a p-value of 0.022. However, with so many comparisons one would expect some to be ‘significant’ (with a p-value of 0.05 or less) just by chance. To account for all these tests the authors should apply some type of correction such as Bonferroni’s adjustment.

Figures are carefully prepared and informative.

Reviewer #2: While the manuscript presents relevant results, there are several issues that impact the clarity and completeness of the analysis. In the INLA-SPDE model for Aedes aegypti egg counts, the variable representing food insecurity appears to be statistically significant, but its origin is unclear, it is not highlighted as other significant variables are, and it is not discussed in the text. Additionally, the reported association with impervious surface coverage (IRR = 0.83; 95% CI: 0.66–1.03) should be carefully reviewed.

The authors state that including LST and mosquito data improves the Chikungunya model's performance, but they do not provide comparative metrics to support this claim. This limits the reader’s ability to evaluate whether the inclusion of these variables truly improves model fit.

Furthermore, in Figure 2, which shows a map of seroprevalence, the source of the values is unclear. The methods suggest that the outcome variable is binary (positive/negative), which makes it difficult to interpret the mapped values. This highlights the importance of providing a full model specification and clear explanation of derived outputs to avoid confusion. Recommend: major revision

**********

**Conclusions**

-Are the conclusions supported by the data presented?

-Are the limitations of analysis clearly described?

-Do the authors discuss how these data can be helpful to advance our understanding of the topic under study?

-Is public health relevance addressed?

Reviewer #1: The Discussion is quite basic and has limited reference to other studies. For example, it concludes that control actions should be focused on ‘the peridomestic environment, such as cleanup campaigns and the elimination of breeding sites, which may be effective in reducing vector density in affected areas’. The importance of peridomestic activities is thoroughly well known in the field (see the CDC’s Surveillance and Control of Aedes aegypti and Aedes albopictus in the United States) and is not a novel finding.

There is reference to ‘a study conducted in a vulnerable community in Salvador, Bahia, Brazil, which reported that structural deficiencies in both housing and surrounding streets were associated with prior CHIKV infection’ by Anjos et al.. Given that the submitted article and the paper by Anjos were both considering chikungunya exposure in the same city, are there other similarities or differences that can be discussed here?

There is discussion of a trend between the risk of exposure to chikungunya and the predicted Ae. aegypti egg count. The map shows a large exposure hotspot that did not have particularly high egg counts. Is there anything to discuss here? Perhaps this could be pointed out as an example of where

egg counts do not accurately reflect the true abundance of adults (the vectorial stage).

The temperature profile of the vulnerable communities is interesting, and the authors make an important point about potential increases in risk with climate change. Are there other studies supporting this finding at a sub-city level?

Overall I think the authors need to do more to show how their findings are useful.

Reviewer #2: Although some of the conclusions are supported by the analyses and results presented, one point that stands out is the authors’ claim that fine-scale spatial data on the vector could serve as an indicator for chikungunya transmission. This statement is not supported by the findings of the study. Additionally, the manuscript lacks a discussion on the limitations of the vector sampling, which was conducted over a short period (one week). This is particularly relevant given that Aedes aegypti populations are highly influenced by climatic factors, and the short sampling window may have significantly affected the observed results. Although the authors criticise traditional vector control methods, the practical measures they propose based on their findings remain within the same traditional framework (e.g., yard cleanup and elimination of breeding sites)

**********

**Editorial and Data Presentation Modifications?**

Use this section for editorial suggestions as well as relatively minor modifications of existing data that would enhance clarity. If the only modifications needed are minor and/or editorial, you may wish to recommend “Minor Revision” or “Accept”.

Reviewer #1: Minor comments

Mosquitoes should not be italicized.

Chikungunya should not be spelled with a capital letter except at the start of a sentence.

Line 1: CHIKV refers to the virus, not the disease. It might be clearer to write ‘Chikungunya fever, caused by chikungunya virus (CHIKV), has emerged…’

‘Hotspots for Ae. aegypti breeding.’ Strictly the authors are looking for larval development sites not places where the mosquitoes breed (mate).

In Table 1, a variable is labelled as ‘Race,’ but the Methods describe this as ‘self-reported skin color.’ For consistency and clarity, I recommend the authors use same term (‘self-reported skin color’) in the table.

Reviewer #2: Accept

**********

**Summary and General Comments**

Use this section to provide overall comments, discuss strengths/weaknesses of the study, novelty, significance, general execution and scholarship. You may also include additional comments for the author, including concerns about dual publication, research ethics, or publication ethics. If requesting major revision, please articulate the new experiments that are needed.

Reviewer #1: This study uses a combination of serological assays, mosquito trapping, socioeconomic surveys and land use data to investigate high risk areas of chikungunya exposure in vulnerable communities of Salvador, Brazil. The article is well written, and seeks to identify areas that might require additional vector control activities, particularly in the context of climate change which could lead to increased vector populations. Although the study could prove valuable in this context, I felt that the manuscript led readers to think that there is a lack of studies of this type, and aside from the useful maps it came to some rather expected conclusions.

In the Introduction, why not reference the review by Whiteman et al 2020 (Do socioeconomic factors drive Aedes mosquito vectors and their arboviral diseases: A systematic review of dengue, chikungunya, yellow fever, and Zika virus)? That authors of that review included almost one hundred studies in their analysis, showing the abundance of previous work on this subject.

Reviewer #2: The study aims to identify, in a vulnerable community, the social and environmental factors associated with the distribution of Aedes aegypti and chikungunya. To achieve this, the authors employ a methodological approach integrating primary data from serological investigation, vector monitoring, and information collected through questionnaires and field observations, in addition to environmental data, all at a fine spatial scale. The study also applies robust statistical modelling, which gives the work relevant scientific value.

The authors identify socio-environmental factors related to the distribution of the disease and the vector in the studied community. However, the paper has some limitations that should be considered. Some important information is missing, and this makes it difficult to fully understand the study. Also, the results need to be discussed in more detail.

**********

PLOS authors have the option to publish the peer review history of their article (what does this mean?). If published, this will include your full peer review and any attached files.

If you choose “no”, your identity will remain anonymous but your review may still be made public.

**Do you want your identity to be public for this peer review?**  For information about this choice, including consent withdrawal, please see our Privacy Policy.

Reviewer #1: No

Reviewer #2: No

[NOTE: If reviewer comments were submitted as an attachment file, they will be attached to this email and accessible via the submission site. Please log into your account, locate the manuscript record, and check for the action link "View Attachments". If this link does not appear, there are no attachment files.]

**Figure resubmission:**

**Reproducibility:**

To enhance the reproducibility of your results, we recommend that authors of applicable studies deposit laboratory protocols in protocols.io, where a protocol can be assigned its own identifier (DOI) such that it can be cited independently in the future. Additionally, PLOS ONE offers an option to publish peer-reviewed clinical study protocols. Read more information on sharing protocols at https://plos.org/protocols?utm_medium=editorial-email&utm_source=authorletters&utm_campaign=protocols

Attachment Submitted filename: Comments_Plos_Neglected.docx

# Author comment: Author response to Decision Letter 1

**DOI:** 10.1371/journal.pntd.0014776.r002

27 Feb 2026

Attachment Submitted filename: Response to Reviewers_Plos_Neglected.docx

# Peer review: Decision Letter 1

**DOI:** 10.1371/journal.pntd.0014776.r003

**Authors:** Paul Mireji

11 May 2026

PNTD-D-25-01036R1

Socio-environmental assessment of Aedes aegypti distribution and risk of chikungunya in vulnerable community.

PLOS Neglected Tropical Diseases

Dear Dr. Khalil,

Thank you for submitting your manuscript to PLOS Neglected Tropical Diseases. After careful consideration, we feel that it has merit but does not fully meet PLOS Neglected Tropical Diseases's publication criteria as it currently stands. Therefore, we invite you to submit a revised version of the manuscript that addresses the points raised during the review process.

Please submit your revised manuscript within by Jun 10 2026 11:59PM. If you will need more time than this to complete your revisions, please reply to this message or contact the journal office at plosntds@plos.org. When you're ready to submit your revision, log on to https://www.editorialmanager.com/pntd/ and select the 'Submissions Needing Revision' folder to locate your manuscript file.

Please include the following items when submitting your revised manuscript:

* A letter that responds to each point raised by the editor and reviewer(s). You should upload this letter as a separate file labeled 'Response to Reviewers'. This file does not need to include responses to any formatting updates and technical items listed in the 'Journal Requirements' section below.

* A marked-up copy of your manuscript that highlights changes made to the original version. You should upload this as a separate file labeled 'Revised Manuscript with Track Changes'.

* An unmarked version of your revised paper without tracked changes. You should upload this as a separate file labeled 'Manuscript'.

If you would like to make changes to your financial disclosure, competing interests statement, or data availability statement, please make these updates within the submission form at the time of resubmission. Guidelines for resubmitting your figure files are available below the reviewer comments at the end of this letter.

As the corresponding author, your ORCID iD is verified in the submission system and will appear in the published article. PLOS supports the use of ORCID, and we encourage all coauthors to register for an ORCID iD and use it as well. Please encourage your coauthors to verify their ORCID iD within the submission system before final acceptance, as unverified ORCID iDs will not appear in the published article. Only the individual author can complete the verification step; PLOS staff cannot verify ORCID iDs on behalf of authors.

We look forward to receiving your revised manuscript.

Kind regards,

Paul O. Mireji, PhD

Section Editor

PLOS Neglected Tropical Diseases

Paul Mireji

Section Editor

PLOS Neglected Tropical Diseases

Shaden Kamhawi

co-Editor-in-Chief

PLOS Neglected Tropical Diseases

orcid.org/0000-0003-4304-636XX

Paul Brindley

co-Editor-in-Chief

PLOS Neglected Tropical Diseases

orcid.org/0000-0003-1765-0002

**Journal Requirements:**

1) We have noticed that you have uploaded Supporting Information files, but you have not included a complete list of legends. Please add a full list of legends for your Supporting Information files ("Database_CHIKV_MR.csv and "Database_ovitraps.xlsx") after the references list.

Note: If the reviewer comments include a recommendation to cite specific previously published works, please review and evaluate these publications to determine whether they are relevant and should be cited. There is no requirement to cite these works unless the editor has indicated otherwise.

**Reviewers' Comments:**

**Comments to the Authors:**

**Please note that one review is uploaded as an attachment.**

Reviewer's Responses to Questions

**Key Review Criteria Required for Acceptance?**

As you describe the new analyses required for acceptance, please consider the following:

**Methods**

-Are the objectives of the study clearly articulated with a clear testable hypothesis stated?

-Is the study design appropriate to address the stated objectives?

-Is the population clearly described and appropriate for the hypothesis being tested?

-Is the sample size sufficient to ensure adequate power to address the hypothesis being tested?

-Were correct statistical analysis used to support conclusions?

-Are there concerns about ethical or regulatory requirements being met?

Reviewer #1: (No Response)

Reviewer #2: All comments are provided in the attached file, with references to the corresponding line numbers in the PDF shared for review.

**********

**Results**

-Does the analysis presented match the analysis plan?

-Are the results clearly and completely presented?

-Are the figures (Tables, Images) of sufficient quality for clarity?

Reviewer #1: (No Response)

Reviewer #2: All comments are provided in the attached file, with references to the corresponding line numbers in the PDF shared for review.

**********

**Conclusions**

-Are the conclusions supported by the data presented?

-Are the limitations of analysis clearly described?

-Do the authors discuss how these data can be helpful to advance our understanding of the topic under study?

-Is public health relevance addressed?

Reviewer #1: (No Response)

Reviewer #2: All comments are provided in the attached file, with references to the corresponding line numbers in the PDF shared for review.

**********

**Editorial and Data Presentation Modifications?**

Use this section for editorial suggestions as well as relatively minor modifications of existing data that would enhance clarity. If the only modifications needed are minor and/or editorial, you may wish to recommend “Minor Revision” or “Accept”.

Reviewer #1: (No Response)

Reviewer #2: (No Response)

**********

**Summary and General Comments**

Use this section to provide overall comments, discuss strengths/weaknesses of the study, novelty, significance, general execution and scholarship. You may also include additional comments for the author, including concerns about dual publication, research ethics, or publication ethics. If requesting major revision, please articulate the new experiments that are needed.

Reviewer #1: The authors have carefully addressed the comments from the original review.

A few minor points: In the their response to reviewer comments, the authors state that "We have clarified in the revised manuscript that further studies specifically designed to analyze incidence data, with tighter temporal alignment between entomological and epidemiological measurements and reduced external interference, are needed to better assess the utility of finescale egg count–based metrics for identifying areas of higher transmission risk. However, I cannot find this reference to incidence data.

References are provided later in the Discussion but I feel line 370 requires a reference.

The authors should carefull check for consistent use of italics (line 367) and abbreviation of Aedes genus name (line 391).

Reviewer #2: As the coordinates are included in the supplementary material (Database_CHIKV_MR), I am concerned about the potential identification of participants’ households.

**********

PLOS authors have the option to publish the peer review history of their article (what does this mean?). If published, this will include your full peer review and any attached files.

If you choose “no”, your identity will remain anonymous but your review may still be made public.

**Do you want your identity to be public for this peer review?**  For information about this choice, including consent withdrawal, please see our Privacy Policy.

Reviewer #1: No

Reviewer #2: No

[NOTE: If reviewer comments were submitted as an attachment file, they will be attached to this email and accessible via the submission site. Please log into your account, locate the manuscript record, and check for the action link "View Attachments". If this link does not appear, there are no attachment files.]

**Figure resubmission:**

**Reproducibility:**

To enhance the reproducibility of your results, we recommend that authors of applicable studies deposit laboratory protocols in protocols.io, where a protocol can be assigned its own identifier (DOI) such that it can be cited independently in the future. Additionally, PLOS ONE offers an option to publish peer-reviewed clinical study protocols. Read more information on sharing protocols at https://plos.org/protocols?utm_medium=editorial-email&utm_source=authorletters&utm_campaign=protocols

Attachment Submitted filename: Review R1_PLOS_R1_.docx

# Author comment: Author response to Decision Letter 2

**DOI:** 10.1371/journal.pntd.0014776.r004

25 Jun 2026

Attachment Submitted filename: Response to Reviewers.docx

# Editor report: Decision Letter 2

**DOI:** 10.1371/journal.pntd.0014776.r005

**Authors:** Paul Mireji, Clarence Mang'era

21 Jul 2026

Response to Reviewers Revised Manuscript with Track Changes Manuscript

Shaden Kamhawi

co-Editor-in-Chief

PLOS Neglected Tropical Diseases

orcid.org/0000-0003-4304-636XX

Paul Brindley

co-Editor-in-Chief

PLOS Neglected Tropical Diseases

orcid.org/0000-0003-1765-0002

**Additional Editor Comments (if provided):**

Revise the response to reviewer 2 on WAIC/model-selection explanation. The phrase "effects ... consistent with current biological understanding" could be interpreted as selecting a model partly because the direction of the result conformed to expectations. This premise risks appearing post hoc. Make it clear in the MS that biological plausibility informed candidate model specification or interpretation, whereas final selection considered predictive fit and parsimony without preferentially selecting models because coefficients had expected directions.

**Reviewers' comments:** **Figure resubmission:**

**Reproducibility:**

To enhance the reproducibility of your results, we recommend that authors of applicable studies deposit laboratory protocols in protocols.io, where a protocol can be assigned its own identifier (DOI) such that it can be cited independently in the future. Additionally, PLOS ONE offers an option to publish peer-reviewed clinical study protocols. Read more information on sharing protocols at https://plos.org/protocols?utm_medium=editorial-email&utm_source=authorletters&utm_campaign=protocols

# Author comment: Author response to Decision Letter 3

**DOI:** 10.1371/journal.pntd.0014776.r006

30 Jul 2026

Attachment Submitted filename: 2026-07-23-Response to Reviewers.pdf

# Editor report: Decision Letter 3

**DOI:** 10.1371/journal.pntd.0014776.r007

**Authors:** Paul Mireji, Clarence Mang'era

22 Sep 2026

Dear Mr. Khalil,

We are pleased to inform you that your manuscript 'Socio-environmental assessment of Aedes aegypti distribution and risk of chikungunya in vulnerable community.' has been provisionally accepted for publication in PLOS Neglected Tropical Diseases.

Before your manuscript can be formally accepted you will need to complete some formatting changes, which you will receive in a follow up email. A member of our team will be in touch with a set of requests.

Please note that your manuscript will not be scheduled for publication until you have made the required changes, so a swift response is appreciated.

IMPORTANT: The editorial review process is now complete. PLOS will only permit corrections to spelling, formatting or significant scientific errors from this point onwards. Requests for major changes, or any which affect the scientific understanding of your work, will cause delays to the publication date of your manuscript.

Should you, your institution's press office or the journal office choose to press release your paper, you will automatically be opted out of early publication. We ask that you notify us now if you or your institution is planning to press release the article. All press must be co-ordinated with PLOS.

Thank you again for supporting Open Access publishing; we are looking forward to publishing your work in PLOS Neglected Tropical Diseases.

Best regards,

Clarence Mang'era, PhD

Academic Editor

PLOS Neglected Tropical Diseases

Paul Mireji

Section Editor

PLOS Neglected Tropical Diseases

Shaden Kamhawi

co-Editor-in-Chief

PLOS Neglected Tropical Diseases

orcid.org/0000-0003-4304-636XX

Paul Brindley

co-Editor-in-Chief

PLOS Neglected Tropical Diseases

orcid.org/0000-0003-1765-0002

***********************************************************

Thank you for addressing the reviewers’ comments through successive revisions. Take note of these grammatical and language errors:

Please complete a thorough language check. Examples include “favor the vector proliferation” (34–35), “households experiencing” (48), “information … is summarized” (134–135), “association … highlights” (303–305), “trends … are … warrant” (349–352), and “estimates … provide” (436–438).

Use “ovitrap results,” “per ovitrap,” and “ovitrap location” (145–150). Rewrite the incomplete sentence at 250–252 and the run-on climate description at 451–456. Replace “a few ten of meters” with “a few tens of meters” (256), “and similar by” with “and similarly by” (259–261), “related with” with “associated with” (345–346), and “susceptibility to floodings” with “flood susceptibility” (509). Remove the repeated “then” (507), correct “The criteria … was” to “The criterion … was” (523–524), and insert the missing space in “package.Spatial” (590). Correct “Plasted wall” and “Not yard” (did you mean "No Yard"?) in the tables. COrrect the duplicated text on lines 611–615, which repeat the paragraph at 606–610.

Reconcile lines 145–147. The reported 134 positive traps conflict with the subgroup total of 138 (56 + 82), and 82/95 equals 86.3%, not 82.1%. Verify the underlying counts and correct all affected totals, percentages and summaries.

Lastly, confirm how eggs were identified as Ae. aegypti, how variable trap exposure durations were handled (550–555). Distinguish parental or guardian consent from children’s assent (469–471).

# Editor report: Acceptance letter

**DOI:** 10.1371/journal.pntd.0014776.r008

**Authors:** Paul Mireji, Clarence Mang'era

Dear Mr. Khalil,

We are delighted to inform you that your manuscript, "Socio-environmental assessment of Aedes aegypti distribution and risk of chikungunya in vulnerable community.," has been formally accepted for publication in PLOS Neglected Tropical Diseases.

We have now passed your article onto the PLOS Production Department who will complete the rest of the publication process. All authors will receive a confirmation email upon publication.

The corresponding author will soon be receiving a typeset proof for review, to ensure errors have not been introduced during production. Please review the PDF proof of your manuscript carefully, as this is the last chance to correct any scientific or type-setting errors. Please note that major changes, or those which affect the scientific understanding of the work, will likely cause delays to the publication date of your manuscript. Note: Proofs for Front Matter articles (Editorial, Viewpoint, Symposium, Review, etc...) are generated on a different schedule and may not be made available as quickly.

Soon after your final files are uploaded, the early version of your manuscript will be published online unless you opted out of this process. The date of the early version will be your article's publication date. The final article will be published to the same URL, and all versions of the paper will be accessible to readers.

For Research Articles, you will receive an invoice from PLOS for your publication fee after your manuscript has reached the completed accept phase. If you receive an email requesting payment before acceptance or for any other service, this may be a phishing scheme. Learn how to identify phishing emails and protect your accounts at https://explore.plos.org/phishing.

Thank you again for supporting open-access publishing; we are looking forward to publishing your work in PLOS Neglected Tropical Diseases.

Best regards,

Shaden Kamhawi

co-Editor-in-Chief

PLOS Neglected Tropical Diseases

Paul Brindley

co-Editor-in-Chief

PLOS Neglected Tropical Diseases
